# A fast continuous time approach for non-smooth convex optimization using Tikhonov regularization technique

**DOI:** 10.1007/s10589-023-00536-6

**Published:** 2023-10-25

**Authors:** Mikhail A. Karapetyants

**Affiliations:** Faculty of Mathematics, University of Vienna, Oskar-Morgenstern-Platz 1, 1090 Vienna, Austria

**Keywords:** Nonsmooth convex optimization, Damped inertial dynamics, Hessian-driven damping, Moreau envelope, Proximal operator, Tikhonov regularization, Strong convergence, 37N40, 46N10, 49M99, 65K05, 65K10, 90C25

## Abstract

In this paper we would like to address the classical optimization problem of minimizing a proper, convex and lower semicontinuous function via the second order in time dynamics, combining viscous and Hessian-driven damping with a Tikhonov regularization term. In our analysis we heavily exploit the Moreau envelope of the objective function and its properties as well as Tikhonov regularization properties, which we extend to a nonsmooth case. We introduce the setting, which at the same time guarantees the fast convergence of the function (and Moreau envelope) values and strong convergence of the trajectories of the system to a minimal norm solution—the element of the minimal norm of all the minimizers of the objective. Moreover, we deduce the precise rates of convergence of the values for the particular choice of parameters. Various numerical examples are also included as an illustration of the theoretical results.

## Introduction

### The formulation of the problem

In the Hilbert space *H*, endowed with the inner product $\langle \cdot , \cdot \rangle$ and the norm $\Vert \cdot \Vert = \sqrt{\langle \cdot , \cdot \rangle }$, we consider the classical minimization problem

$$\begin{aligned} \min _{x \in H} \Phi (x) \end{aligned}$$

of a proper, convex and lower semicontinuous function $\Phi$. In order to address this question we would like to use the well-known technique of linking the gradient of the Moreau envelope $\Phi _\lambda$ of the objective function $\Phi$ to the second order in time differential equation

1 $$\begin{aligned} \ddot{x}(t) + \alpha \sqrt{\varepsilon (t)} \dot{x}(t) + \beta \frac{d}{dt} \left( \nabla \Phi _{\lambda (t)} (x(t)) \right) + \nabla \Phi _{\lambda (t)} (x(t)) + \varepsilon (t) x(t) = 0 \text { for } t \ge t_0, \end{aligned}$$

and study its convergence properties, showing that alongside the trajectory—the solution of (1)—the function $\Phi$ converges to its minimum. The initial conditions are $x(t_0) = x_0 \in H$ and $\dot{x}(t_0) = x_1 \in H$ and $\alpha , \beta \text { and } t_0 > 0$, $\Phi : H \longrightarrow \overline{\mathbb {R}} = \mathbb {R} \cup \{ \pm \infty \}$ is a proper, convex and lower semicontinuous function and $\Phi _\lambda$ is its Moreau envelope of the index $\lambda > 0$. The function $\lambda : [t_0, +\infty ) \longrightarrow \mathbb {R}_+$ is assumed to be continuously differentiable and nondecreasing while the function $\varepsilon : [t_0, +\infty ) \longrightarrow \mathbb {R}_+$ is continuously differentiable and nonincreasing with the property $\lim _{t \rightarrow +\infty } \varepsilon (t) = 0$. In addition, we assume that $\mathop {\textrm{argmin}}\limits \Phi$, which is the set of global minimizers of $\Phi$, is not empty and denote by $\Phi ^*$ the optimal objective value of $\Phi$. Finally, for every $t \ge t_0$ let us introduce the strongly convex function $\varphi _{\varepsilon (t), \lambda (t)}: H \longrightarrow \mathbb {R}$ defined as $\varphi _{\varepsilon (t), \lambda (t)} (x) = \Phi _{\lambda (t)}(x) + \frac{\varepsilon (t) \Vert x \Vert ^2}{2}$, and let us denote the unique minimizer of $\varphi _{\varepsilon (t), \lambda (t)}$ as $x_{\varepsilon (t), \lambda (t)} = \mathop {\textrm{argmin}}\limits _{H} \varphi _{\varepsilon (t), \lambda (t)}$.

The main goal of the research is to provide the setting in a nonsmooth case, where we would have fast convergence of the function values combined with strong convergence of the trajectories to the element of the minimal norm from the set of all minimizers of the objective function. This analysis is an extrapolation of the one conducted in [3] to the case of a nonsmooth objective function. We also aim to provide the exact rates of convergence of the values for the polynomial choice of the smoothing parameter $\lambda$ and Tikhonov function $\varepsilon$. As a conclusion, multiple numerical experiments were conducted allowing better understanding of the theoretical results.

### Related results

The Moreau envelope plays a significant role in nonsmooth optimization. It is defined as ($\Phi : H \rightarrow \overline{\mathbb {R}}$ is a proper, convex and lower semicontinuous function)

$$\begin{aligned} \Phi _\lambda : H \rightarrow \mathbb {R}, \quad \Phi _{\lambda } (x) \ = \ \inf _{y \in H} \left\{ \Phi (y) + \frac{1}{2 \lambda } \Vert x - y \Vert ^2 \right\} , \end{aligned}$$

where $\lambda > 0$. $\Phi _\lambda$ is convex and continuously differentiable with

2 $$\begin{aligned} \nabla \Phi _{\lambda } (x) = \frac{1}{\lambda } ( x - \mathop {\textrm{prox}}\limits \nolimits _{\lambda \Phi } (x)) \quad \forall x \in H, \end{aligned}$$

and $\nabla \Phi _\lambda$ is $\frac{1}{\lambda }$-Lipschitz continuous. Here,

$$\begin{aligned} \mathop {\textrm{prox}}\limits \nolimits _{\lambda \Phi }: H \rightarrow H, \quad \mathop {\textrm{prox}}\limits \nolimits _{\lambda \Phi } (x) = \mathop {\textrm{argmin}}\limits _{y \in H} \left\{ \Phi (y) + \frac{1}{2 \lambda } \Vert x - y \Vert ^2 \right\} \end{aligned}$$

denotes the proximal operator of $\Phi$ of parameter $\lambda$. Moreover (see [1]),

3 $$\begin{aligned} \frac{d}{dt} \Phi _{\lambda (t)} (x) = - \frac{{\dot{\lambda }}(t)}{2} \Vert \nabla \Phi _{\lambda (t)} (x) \Vert ^2 \ \forall x \in H. \end{aligned}$$

The work [5] by Attouch-László serves as a starting point for a lot of different research topics in nonsmooth optimization. The following dynamics was considered

4 $$\begin{aligned} \ddot{x}(t) + \frac{\alpha }{t} \dot{x}(t) + \beta \frac{d}{dt} \nabla \Phi _{\lambda (t)}(x(t)) + \nabla \Phi _{\lambda (t)}(x(t)) = 0 \end{aligned}$$

where $\alpha > 1$ and $\beta > 0$, and the term $\frac{d}{dt} \nabla \Phi _{\lambda (t)}(x(t))$ is inspired by the Hessian driven damping term in the case of smooth functions. For this system multiple fundamental results were proven, such as convergence rates for the Moreau envelope values as well as for the velocity of the system

$$\begin{aligned} \Phi _{\lambda (t)}(x(t)) - \Phi ^* = o\left( \frac{1}{t^2} \right) \text { and } \Vert \dot{x}(t) \Vert = o\left( \frac{1}{t} \right) \text { as } t \rightarrow +\infty , \end{aligned}$$

from where convergence rates for the $\Phi$ along the trajectories themselves were deduced

$$\begin{aligned}{} & {} \Phi \big ( \mathop {\textrm{prox}}\limits \nolimits _{\lambda (t) \Phi } (x(t)) \big ) - \Phi ^* = o\left( \frac{1}{t^2} \right) \text { and } \\ {}{} & {} \Vert \mathop {\textrm{prox}}\limits \nolimits _{\lambda (t) \Phi } (x(t)) - x(t) \Vert = o\left( \frac{\sqrt{\lambda (t)}}{t} \right) \text { as } t \rightarrow +\infty . \end{aligned}$$

In addition, convergence rates for the gradient of the Moreau envelope of parameter $\lambda (t)$ and its time derivative along *x*(*t*) were established

$$\begin{aligned} \Vert \nabla \Phi _{\lambda (t)} (x(t)) \Vert = o\left( \frac{1}{t^2} \right) \text { and } \left\| \frac{d}{dt} \nabla \Phi _{\lambda (t)} (x(t)) \right\| = o\left( \frac{1}{t^2} \right) \text { as } t \rightarrow +\infty . \end{aligned}$$

Moreover, the weak convergence of the trajectories *x*(*t*) to a minimizer of $\Phi$ as $t \rightarrow +\infty$ was deduced.

From here one may go in many directions in order to continue investigating the topic of second order dynamics. Time scaling, for instance, can be introduced to improve the speed of convergence of the values, as it was done in [12]. Another way to proceed is to consider the so-called Tikhonov regularization technique, to which we devote the next few pages of our manuscript.

The presence of the Tikhonov term in the system equation dramatically influences the behaviour of its trajectories, namely, under some appropriate conditions, it improves the convergence of the trajectories from weak to a strong one. Not only that, but it also ensures the convergence not to an arbitrary element from the set of all minimizers of the objective, but to the particular one, which has the smallest norm. Under the presence of the Tikhonov term in the system it is still possible to obtain fast rates of convergence of the function values. Systems with Tikhonov regularization were studied in, for instance, in [2–4, 6, 10, 11, 13, 14].

One of the fine examples in a smooth setting is presented below (see [3])

$$\begin{aligned} \ddot{x}(t) + \alpha \sqrt{\varepsilon (t)} \dot{x}(t) + \beta \frac{d}{dt} \Big ( \nabla \varphi _t (x(t)) + (p - 1) \varepsilon (t) x(t) \Big ) + \nabla \varphi _t (x(t)) = 0 \text { for } t \ge t_0, \end{aligned}$$

where $\varphi _t (x) = \Phi (x) + \frac{\varepsilon (t) \Vert x \Vert ^2}{2}$, $\Phi : H \longrightarrow \mathbb {R}$ is twice continuously differentiable and convex, $\varepsilon$ is nonincreasing and goes to zero, as $t \rightarrow +\infty$, and *p* is chosen appropriately. This system inherits the properties of fast convergence rates of the function values, being of the order $\frac{1}{t^2}$, and additionally provides the strong convergence results for the trajectories of the system in the same setting.

Concerning the nonsmooth case we refer to [11], where it was covered for the more general systems, governed by a maximally monotone operator, but with a different damping. The authors studied the following dynamics

5 $$\begin{aligned} \ddot{x}(t) + \frac{\alpha }{t^q} \dot{x}(t) + \beta \frac{d}{dt} \Big ( A_{\lambda (t)} (x(t)) \Big ) + A_{\lambda (t)} (x(t)) + \varepsilon (t) x(t) = 0 \text { for } t \ge t_0, \end{aligned}$$

where $\alpha > 0$, $\beta \ge 0$, $0 < q \le 1$ and $\lambda (t) = \lambda t^{2q}$ for $\lambda > 0$, *A* is a maximally monotone operator and $A_\lambda$ is its Yosida regularization of the order $\lambda$. The system (5) is related to the inclusion problem $0 \in Ax$. The authors showed the fast convergence rates for $\Vert \dot{x}(t) \Vert$, $\Vert A_{\lambda (t)} (x(t)) \Vert$ and $\Vert \frac{d}{dt} A_{\lambda (t)} (x(t)) \Vert$ being of the order $\frac{1}{t^q}$, $\frac{1}{t^{2q}}$ and $\frac{1}{t^{3q}}$ correspondingly. Moreover, they established the strong convergence of the trajectories of the system. In the section 4 of [11] the authors also considered a very interesting particular case of $A = \partial \Phi$ using the well-known connection $A_\lambda = \left( \partial \Phi \right) _\lambda = \nabla \Phi _\lambda$ for all $\lambda > 0$. In this connection we would like to formulate the next remark.

#### Remark 1

We would like to stress that Theorem 11 of [11] does not cover the case presented in this paper.

1. First of all, the systems (1) and (5) have different damping coefficients. The damping in (1) depends on the Tikhonov function $\varepsilon$, while the damping in (5) is taken in a polynomial form $\frac{1}{t^q}$. Thus, if we take $\varepsilon (t) = \frac{1}{t^{2q}}$ in (5) to mimic the relation between the damping parameter and the Tikhonov function as in (1), then one of the conditions of Theorem 11 becomes
  $$\begin{aligned} \int _{t_0}^{+\infty } t^{3q} \varepsilon ^2(t) dt \ = \ \int _{t_0}^{+\infty } \frac{1}{t^q} dt \ < \ +\infty , \end{aligned}$$
  where $0< q < 1$, which is obviously not fulfilled.
2. Secondly, the smoothing parameter $\lambda$ in [11] is fixed, while our analysis holds for more general choice of $\lambda$. However, if we want to consider the polynomial case of parameters (Sect. 4), then we indeed arrive at a similar restriction for $\lambda$: in Sect. 4 we will discover that for strong convergence of the trajectories and polynomial choice of parameters, $\lambda (t) = t^l$, we have to take $0 \le l < 2$, which is a wider range than $0< q < 1$ for $\lambda (t) = t^{2q}$.
3. Finally, the sets of conditions $\left( C_0 \right)$–$\left( C_4 \right)$ and our assumptions (11)–(14) lead to different settings in case of polynomial choice of the function $\varepsilon (t) = \frac{1}{t^d}$, $d > 0$ (Sect. 4). Namely, according to Corollary 9 of [11] the setting to satisfy all the conditions in the analysis in this case for $\beta > 0$ is $\max \left\{ 1-q, \frac{3q + 1}{2} \right\} < d \le 1 + q$ with $0< q < 1$, whereas as we will see later (Sect. 4) our set of conditions allows $1 \le d \le 2$, which is more flexible in terms of the upper bound while being almost the same in terms of the lower bound. Thus, $d = 2$ is not an option in [11], but the lower limitation for the choice of *d* could be wider depending on the choice of *q*. Since the rates of convergence are better for the bigger values of *d* (Theorem 8), this additional flexibility of the upper bound justifies, in our opinion, the restrictions for the lower one.

In this paper we aim to develop the ideas presented in [3] for $p = 0$ to cover the nonsmooth case. The objective function $\Phi$ is no longer required to be (continuously) differentiable, which gives us more freedom in choosing the latter. Moreover, we show that the main quantities $\Phi _{\lambda (t)} (x(t)) - \Phi ^*$, $\Phi \left( \mathop {\textrm{prox}}\limits _{\lambda (t) \Phi } (x(t)) \right) - \Phi ^*$, $\Vert \mathop {\textrm{prox}}\limits _{\lambda (t) \Phi } (x(t)) - x(t) \Vert$ and $\Vert x(t) - x_{\varepsilon (t), \lambda (t)} \Vert$ go to zero, as $t \rightarrow +\infty$, without specifying (as it was done in [3]) the choice of the functions $\varepsilon$ and $\lambda$. We are also able to obtain rates of convergence of function values in case of the polynomial choice of parameters $\varepsilon (t) = \frac{1}{t^d}$ for $d = 2$, which is not an option in [3].

### Our contribution

Our main focus throughout this manuscript is obtaining fast convergence of function values alongside with the strong convergence of the trajectories to a minimal norm solution. The main result is given by the Theorem 5, namely, for any $t \ge t_0$

$$\begin{aligned}{} & {} \Phi _{\lambda (t)} (x(t)) - \Phi ^* \ \le \ E(t) + \frac{\varepsilon (t)}{2} \Vert x^* \Vert ^2,\\{} & {} \Phi \left( \mathop {\textrm{prox}}\limits \nolimits _{\lambda (t) \Phi }(x(t)) \right) - \Phi ^* \ \le \ E(t) + \frac{\varepsilon (t)}{2} \Vert x^* \Vert ^2,\\{} & {} \Vert \mathop {\textrm{prox}}\limits \nolimits _{\lambda (t) \Phi } (x(t)) - x(t) \Vert ^2 \ \le \ 2\lambda (t) E(t) + \lambda (t)\varepsilon (t) \Vert x^* \Vert ^2 \end{aligned}$$

and

$$\begin{aligned} \Vert x(t) - x_{\varepsilon (t), \lambda (t)} \Vert ^2 \ \le \ \frac{2 E(t)}{\varepsilon (t)} \end{aligned}$$

and, as we will see later (Theorem 7), all the quantities on the right-hand side of the inequalities are going to zero, as $t \rightarrow +\infty$, under some additional, yet not very restrictive, assumptions.

A rather interesting particular case of polynomial parameters ($\varepsilon (t) = \frac{1}{t^d}$, $\lambda (t) = t^l$, $l, d > 0$) is also covered in this paper which gives us the following precise rates of convergence. For *t* large enough we deduce

$$\begin{aligned}{} & {} \Phi _{\lambda (t)} (x(t)) - \Phi ^* \ \le \ \frac{1}{t^d},\\{} & {} \Phi \left( \mathop {\textrm{prox}}\limits \nolimits _{\lambda (t) \Phi }(x(t)) \right) - \Phi ^* \ \le \ \frac{1}{t^d},\\{} & {} \Vert \mathop {\textrm{prox}}\limits \nolimits _{\lambda (t) \Phi } (x(t)) - x(t) \Vert ^2 \ \le \ \frac{1}{t^{\frac{d}{2} + 1 - l}} \end{aligned}$$

and

$$\begin{aligned} \Vert x(t) - x_{\varepsilon (t), \lambda (t)} \Vert ^2 \ \le \ \frac{1}{t^{1-\frac{d}{2}}}, \end{aligned}$$

where $1 \le d < 2$ and $0 \le l < d$. The state-of-the-art rates of convergence of the function values are of the order $\frac{1}{t^2}$, and since *d* is assumed to be strictly less than 2, we obtain almost as good as state-of-the-art estimates.

Finally, the special case of $d = 2$ is also considered in this manuscript, which gives the following results depending on the value of the damping coefficient $\alpha$.

1. If $0< \alpha < 2$, then for *t* large enough
  $$\begin{aligned}{} & {} \Phi _{\lambda (t)} (x(t)) - \Phi ^* \ \le \ \frac{1}{t^{\frac{\alpha }{2} + 1}},\\{} & {} \Phi \left( \mathop {\textrm{prox}}\limits \nolimits _{\lambda (t) \Phi }(x(t)) \right) - \Phi ^* \ \le \ \frac{1}{t^{\frac{\alpha }{2} + 1}} \end{aligned}$$
  and
  $$\begin{aligned} \Vert \mathop {\textrm{prox}}\limits \nolimits _{\lambda (t) \Phi } (x(t)) - x(t) \Vert ^2 \ \le \ \frac{1}{t^{\frac{\alpha }{2} - l + 1}}. \end{aligned}$$
2. If $\alpha \ge 2$, then for *t* large enough
  $$\begin{aligned}{} & {} \Phi _{\lambda (t)} (x(t)) - \Phi ^* \ \le \ \frac{1}{t^2},\\{} & {} \Phi \left( \mathop {\textrm{prox}}\limits \nolimits _{\lambda (t) \Phi }(x(t)) \right) - \Phi ^* \ \le \ \frac{1}{t^2} \end{aligned}$$
  and
  $$\begin{aligned} \Vert \mathop {\textrm{prox}}\limits \nolimits _{\lambda (t) \Phi } (x(t)) - x(t) \Vert ^2 \ \le \ \frac{1}{t^{2-l}}. \end{aligned}$$

In this case we cannot guarantee the strong convergence of the trajectories, but for $\alpha \ge 2$ (which is often the choice of the damping parameter in the literature) we show the best known rates of convergence.

The paper is structured as follows. Section 2 gathers some preliminary results, which we will need in our analysis. The main results of our research are presented in Sect. 3. Section 4 provides the polynomial setting, in which the results are valid and the analysis works and establishes the actual rates of convergence of the values and the trajectories. Finally, Sect. 5 is all about numerical experiments, which illustrate the theory.

## Preliminaries

### Auxiliary estimates and properties

Let us begin with introducing the so-called first order optimality condition, as we will require it later in our analysis. In our case it reads as

6 $$\begin{aligned} \nabla \Phi _{\lambda (t)} (x_{\varepsilon (t), \lambda (t)}) + \varepsilon (t) x_{\varepsilon (t), \lambda (t)} = 0. \end{aligned}$$

Now we continue with the following lemma (see [9], Proposition 12.22, for the first term of the lemma and [7], Appendix, A1, for the second one).

#### Lemma 1

Let $\Phi : H \longrightarrow \overline{\mathbb {R}}$ be a proper, convex and lower semicontinuous function, $\lambda , \mu > 0$. Then

1. $$(\Phi _\lambda )_\mu = \Phi _{\lambda + \mu }$$.
2. $$ \mathop {\textrm{prox}}\limits _{\mu \Phi _\lambda } = \frac{\lambda }{\lambda + \mu } \mathop {\textrm{Id}}\limits + \frac{\mu }{\lambda + \mu } \mathop {\textrm{prox}}\limits _{(\lambda + \mu ) \Phi } $$.

The following estimates will be used later to evaluate the derivative of our energy function.

#### Lemma 2

The following properties are satisfied:

1. for each $$t \ge t_0$$, $$\frac{d}{dt} \left( \varphi _{\varepsilon (t), \lambda (t)} (x_{\varepsilon (t), \lambda (t)}) \right) = \frac{1}{2} \left( {\dot{\varepsilon }}(t) - \dot{\lambda }(t) \varepsilon ^2(t) \right) \Vert x_{\varepsilon (t), \lambda (t)} \Vert ^2$$;
2. the function $t \mapsto x_{\varepsilon (t), \lambda (t)}$ is Lipschitz continuous on the compact intervals of $(t_0, +\infty )$, thus, is almost everywhere differentiable. Moreover, for almost every $t \ge t_0$
  $$\begin{aligned} \left( \frac{2 {\dot{\lambda }}(t)}{\lambda (t)} - \frac{\dot{\varepsilon }(t)}{\varepsilon (t)} \right) \Vert x_{\varepsilon (t), \lambda (t)} \Vert \ge \left\| \frac{d}{dt} x_{\varepsilon (t), \lambda (t)} \right\| . \end{aligned}$$

Let us also mention two key properties of the Tikhonov regularization, which we will use later in the analysis. For the next Lemma see also Proposition 5 of [11].

#### Lemma 3

Suppose that

7 $$\begin{aligned} \lim _{t \rightarrow +\infty } \lambda (t) \varepsilon (t) \ = \ 0. \end{aligned}$$

Then the following properties of the mapping $t \longrightarrow x_{\varepsilon (t), \lambda (t)}$ are satisfied:

8 $$\begin{aligned} \text { for } x^* = \mathop {\textrm{proj}}\limits \nolimits _{\mathop {\textrm{argmin}}\limits \Phi }(0), \ \Vert x_{\varepsilon (t), \lambda (t)} \Vert \le \Vert x^* \Vert \text { for all } t \ge t_0 \end{aligned}$$

and

9 $$\begin{aligned} \lim _{t \rightarrow +\infty } \Vert x_{\varepsilon (t), \lambda (t)} - x^* \Vert = 0. \end{aligned}$$

Lemmas 2 and 3 will be rigorously proven in the Appendix.

### Existence and uniqueness of the solution of (1)

Our nearest goal is to deduce the existence and uniqueness of the solution of the dynamical system (1). Suppose $\beta > 0$. Let us integrate (1) from $t_0$ to *t* to obtain

$$\begin{aligned}{} & {} \dot{x}(t) + \beta \nabla \Phi _{\lambda (t)} (x(t)) + \int _{t_0}^t \left( \alpha \sqrt{\varepsilon (s)} \dot{x}(s) + \nabla \Phi _{\lambda (s)} (x(s)) + \varepsilon (s) x(s) \right) ds \\ {}{} & {} - \dot{x}(t_0) - \beta \nabla \Phi _{\lambda (t_0)} (x(t_0)) \ = \ 0. \end{aligned}$$

Denoting $z(t):= \int _{t_0}^t \left( \alpha \sqrt{\varepsilon (s)} \dot{x}(s) + \nabla \Phi _{\lambda (s)} (x(s)) + \varepsilon (s) x(s) \right) ds - \big ( \dot{x}(t_0) + \beta \nabla \Phi _{\lambda (t_0)} (x_0)) \big )$ for every $t \ge t_0$ and noticing that $\dot{z}(t) = \alpha \sqrt{\varepsilon (t)} \dot{x}(t) + \nabla \Phi _{\lambda (t)} (x(t)) + \varepsilon (t) x(t)$ we deduce, that (1) is equivalent to

$$\begin{aligned} {\left\{ \begin{array}{ll} \dot{x}(t) + \beta \nabla \Phi _{\lambda (t)} (x(t)) + z(t) = 0, \\ \dot{z}(t) - \alpha \sqrt{\varepsilon (t)} \dot{x}(t) - \nabla \Phi _{\lambda (t)}(x(t)) - \varepsilon (t) x(t) = 0, \\ x(t_0) = x_0, \ z(t_0) = -\left( \dot{x}(t_0) + \beta \nabla \Phi _{\lambda (t_0)} (x_0) \right) . \end{array}\right. } \end{aligned}$$

Let us multiply the second one by $\beta$ and then by summing it with the first line we get rid of the gradient of the Moreau envelope in the second equation

$$\begin{aligned} {\left\{ \begin{array}{ll} \dot{x}(t) + \beta \nabla \Phi _{\lambda (t)} (x(t)) + z(t) = 0, \\ \beta \dot{z}(t) + \left( 1 - \alpha \beta \sqrt{\varepsilon (t)} \right) \dot{x}(t) - \beta \varepsilon (t) x(t) + z(t) = 0,\\ x(t_0) = x_0, \ z(t_0) = -\left( \dot{x}(t_0) + \beta \nabla \Phi _{\lambda (t_0)} (x_0) \right) . \end{array}\right. } \end{aligned}$$

We denote now $y(t) = \beta z(t) + \left( 1 - \alpha \beta \sqrt{\varepsilon (t)} \right) x(t)$, and, after simplification, we obtain the following equivalent formulation for the dynamical system

$$\begin{aligned} {\left\{ \begin{array}{ll} \dot{x}(t) + \beta \nabla \Phi _{\lambda (t)} (x(t)) + \left( \alpha \sqrt{\varepsilon (t)} - \frac{1}{\beta } \right) x(t) + \frac{1}{\beta } y(t) = 0, \\ \dot{y}(t) + \left( \frac{\alpha \beta \dot{\varepsilon }(t)}{2\sqrt{\varepsilon (t)}} - \beta \varepsilon (t) - \frac{1}{\beta } + \alpha \sqrt{\varepsilon (t)} \right) x(t) + \frac{1}{\beta } y(t) = 0, \\ x(t_0) = x_0, \ y(t_0) = -\beta \left( \dot{x}(t_0) + \beta \nabla \Phi _{\lambda (t_0)} (x_0) \right) + \left( 1 - \alpha \beta \sqrt{\varepsilon (t_0)} \right) x_0. \end{array}\right. } \end{aligned}$$

In case $\beta = 0$ for every $t \ge t_0$, (1) can be equivalently written as

$$\begin{aligned} {\left\{ \begin{array}{ll} \dot{x}(t) - y(t) = 0, \\ \dot{y}(t) + \alpha \sqrt{\varepsilon (t)} y(t) + \nabla \Phi _{\lambda (t)}(x(t)) + \varepsilon (t) x(t) = 0, \\ x(t_0) = x_0, \ y(t_0) = \dot{x}(t_0). \end{array}\right. } \end{aligned}$$

Therefore, based on the two reformulations of the dynamical system (1) above we provide the following existence and uniqueness result, which is a consequence of the Cauchy-Lipschitz theorem for strong global solutions. The proof follows the lines of the proofs of Theorem 1 in [5] or of Theorem 1.1 in [8] with some small adjustments.

#### Theorem 4

Suppose that there exists $\lambda _0 > 0$ such that $\lambda (t) \ge \lambda _0$ for all $t \ge t_0$. Then for every $(x_0, \dot{x}(t_0)) \in H \times H$ there exists a unique strong global solution $x: [t_0, +\infty ) \mapsto H$ of the continuous dynamics (1) which satisfies the Cauchy initial conditions $x(t_0) = x_0$ and $\dot{x}(t_0) = \dot{x}_0$.

## Abstract convergence results of the function values and strong convergence of the trajectories

This section is devoted to establishing some crucial estimates for the following quantities

$\Phi _{\lambda (t)}(x(t)) - \Phi ^*$ and $\Vert x(t) - x_{\varepsilon (t), \lambda (t)}\Vert$ for all $t \ge t_0$. In order to do so we will use the ideas and methods of Lyapunov analysis. We introduce the energy function

10 $$\begin{aligned} \begin{aligned} E(t) \ = \ {}&\varphi _{\varepsilon (t), \lambda (t)} (x(t)) - \varphi _{\varepsilon (t), \lambda (t)} (x_{\varepsilon (t), \lambda (t)}) \\&+ \ \frac{1}{2} \left\| \gamma \sqrt{\varepsilon (t)} \left( x(t) - x_{\varepsilon (t), \lambda (t)} \right) + \dot{x}(t) + \beta \nabla \Phi _{\lambda (t)} (x(t)) \right\| ^2, \end{aligned} \end{aligned}$$

where $\frac{\alpha }{2} \le \gamma < \alpha$. The next theorem provides the main result of this section.

### Theorem 5

Let $x: [t_0, +\infty ) \longrightarrow H$ be a solution of (1). Then for any $t \ge t_0$

$$\begin{aligned}{} & {} \Phi _{\lambda (t)} (x(t)) - \Phi ^* \ \le \ E(t) + \frac{\varepsilon (t)}{2} \Vert x^* \Vert ^2,\\{} & {} \Phi \left( \mathop {\textrm{prox}}\limits \nolimits _{\lambda (t) \Phi }(x(t)) \right) - \Phi ^* \ \le \ E(t) + \frac{\varepsilon (t)}{2} \Vert x^* \Vert ^2,\\{} & {} \Vert \mathop {\textrm{prox}}\limits \nolimits _{\lambda (t) \Phi } (x(t)) - x(t) \Vert ^2 \ \le \ 2\lambda (t) E(t) + \lambda (t)\varepsilon (t) \Vert x^* \Vert ^2 \end{aligned}$$

and

$$\begin{aligned} \Vert x(t) - x_{\varepsilon (t), \lambda (t)} \Vert ^2 \ \le \ \frac{2 E(t)}{\varepsilon (t)} \end{aligned}$$

and the trajectory *x*(*t*) converges strongly to $x^*$ as soon as $\lim _{t \rightarrow +\infty } \frac{E(t)}{\varepsilon (t)} = 0$.

### Proof

Consider

$$\begin{aligned} \Phi _{\lambda (t)} (x(t)) - \Phi ^*&= \varphi _{\varepsilon (t), \lambda (t)} (x(t)) - \varphi _{\varepsilon (t), \lambda (t)} (x^*) + \frac{\varepsilon (t)}{2} \left( \Vert x^* \Vert ^2 - \Vert x(t) \Vert ^2 \right) \\&= \varphi _{\varepsilon (t), \lambda (t)} (x(t)) - \varphi _{\varepsilon (t), \lambda (t)} (x_{\varepsilon (t), \lambda (t)}) + \varphi _{\varepsilon (t), \lambda (t)} (x_{\varepsilon (t), \lambda (t)}) \\&\quad - \varphi _{\varepsilon (t), \lambda (t)} (x^*) \\&\quad + \frac{\varepsilon (t)}{2} \left( \Vert x^* \Vert ^2 - \Vert x(t) \Vert ^2 \right) \\ {}&\le \varphi _{\varepsilon (t), \lambda (t)} (x(t)) - \varphi _{\varepsilon (t), \lambda (t)} (x_{\varepsilon (t), \lambda (t)}) + \frac{\varepsilon (t)}{2} \left( \Vert x^* \Vert ^2 - \Vert x(t) \Vert ^2 \right) \end{aligned}$$

Using the definition of *E* we obtain

$$\begin{aligned} \Phi _{\lambda (t)} (x(t)) - \Phi ^* \ \le \ E(t) + \frac{\varepsilon (t)}{2} \Vert x^* \Vert ^2. \end{aligned}$$

By the definition of the proximal mapping

$$\begin{aligned}{} & {} \Phi _{\lambda (t)}(x(t)) - \Phi ^* \ = \ \Phi \left( \mathop {\textrm{prox}}\limits \nolimits _{\lambda (t) \Phi }(x(t)) \right) - \Phi ^* + \frac{1}{2\lambda (t)} \Vert \mathop {\textrm{prox}}\limits \nolimits _{\lambda (t) \Phi } (x(t)) \\ {}{} & {} - x(t) \Vert ^2 \quad \forall t \ge t_0. \end{aligned}$$

Thus,

$$\begin{aligned} \Phi \left( \mathop {\textrm{prox}}\limits \nolimits _{\lambda (t) \Phi }(x(t)) \right) - \Phi ^* \ \le \ E(t) + \frac{\varepsilon (t)}{2} \Vert x^* \Vert ^2 \end{aligned}$$

and

$$\begin{aligned} \frac{1}{2\lambda (t)} \Vert \mathop {\textrm{prox}}\limits \nolimits _{\lambda (t) \Phi } (x(t)) - x(t) \Vert ^2 \ \le \ E(t) + \frac{\varepsilon (t)}{2} \Vert x^* \Vert ^2. \end{aligned}$$

The second result immediately follows from the $\varepsilon (t)$-strong convexity of $\varphi _{\varepsilon (t), \lambda (t)}$:

$$\begin{aligned} \varphi _{\varepsilon (t), \lambda (t)} (x(t)) - \varphi _{\varepsilon (t), \lambda (t)} (x_{\varepsilon (t), \lambda (t)}) \ge \frac{\varepsilon (t)}{2} \Vert x(t) - x_{\varepsilon (t), \lambda (t)} \Vert ^2 \end{aligned}$$

and thus

$$\begin{aligned} E(t) \ge \frac{\varepsilon (t)}{2} \Vert x(t) - x_{\varepsilon (t), \lambda (t)} \Vert ^2. \end{aligned}$$

Finally, by $\lim _{t \rightarrow +\infty } \varepsilon (t) = 0$ and (9) we deduce the strong convergence of the trajectories to $x^*$ as soon as $\lim _{t \rightarrow +\infty } \frac{E(t)}{\varepsilon (t)} = 0$. $\square$

Theorem 5 provided some abstract estimates for the important quantities. In order to show that these estimates are actually meaningful, we will have to first estimate the energy functional *E*. The idea is to show that this energy function satisfies the following differential inequality, as it was done in [3],

$$\begin{aligned} \dot{E}(t) + \mu (t) E(t) + \frac{\beta }{2} \Vert \nabla \varphi _{\varepsilon (t), \lambda (t)} (x(t)) \Vert ^2 \ \le \ \frac{g(t) \Vert x^* \Vert ^2}{2} \text { for all } t \ge t_0, \end{aligned}$$

where $\mu (t) = \left( \alpha - \gamma \right) \sqrt{\varepsilon (t)} - \frac{{\dot{\varepsilon }}(t)}{2 \varepsilon (t)}$ and *g* are positive functions. The next theorem provides the analysis needed to obtain the desired inequality.

### Theorem 6

Let $x: [t_0, +\infty ) \longrightarrow H$ be a solution of (1). Assume that (7) holds and suppose that there exist $a, c > 0$ such that for *t* large enough it holds that

11 $$\begin{aligned}&\frac{d}{dt} \left( \frac{1}{\sqrt{\varepsilon (t)}} \right) \ \le \ \min \left\{ 2 \gamma - \alpha - \frac{\gamma \beta \dot{\varepsilon }(t)}{2 \varepsilon (t)}, \ \alpha - \gamma \frac{a + 1}{a} \right\} \end{aligned}$$

12 $$\begin{aligned}&\left( 2 \gamma (\alpha - \gamma ) + \frac{\gamma }{c} - 1 \right) \varepsilon (t) - \beta {\dot{\varepsilon }}(t) \ \le \ 0, \end{aligned}$$

13 $$\begin{aligned}&2 \beta \varepsilon ^2(t) + \left( 2 - \gamma \beta \sqrt{\varepsilon (t)} \right) {\dot{\varepsilon }}(t) \ \le \ 0 \end{aligned}$$

and

14 $$\begin{aligned} \left( \frac{\gamma }{a} + 2 (\alpha - \gamma ) \right) \beta ^2 \sqrt{\varepsilon (t)} - \frac{3 \beta ^2 {\dot{\varepsilon }}(t)}{2 \varepsilon (t)} - {\dot{\lambda }}(t) \ \le \ \beta . \end{aligned}$$

Then there exists $t_1 \ge t_0$ such that for all $t \ge t_1$

$$\begin{aligned} \beta \int _{t_1}^t \Vert \nabla \varphi _{\varepsilon (s)} (x(s)) \Vert ^2 ds \ \le \ 2 E(t_1) + \Vert x^* \Vert ^2 \int _{t_1}^t g(s) ds \end{aligned}$$

and

$$\begin{aligned} E(t) \ \le \ \frac{\Vert x^* \Vert ^2}{2 \Gamma (t)} \int _{t_1}^t \Gamma (s) g(s) ds + \frac{\Gamma (t_1) E(t_1)}{\Gamma (t)}, \end{aligned}$$

where $$\Gamma (t) = \exp \left( \int _{t_1}^t \mu (s) ds \right) $$ and $$g(t) = {\dot{\lambda }}(t) \varepsilon ^2(t) - {\dot{\varepsilon }}(t) + \frac{\gamma \beta {\dot{\varepsilon }}(t) \sqrt{\varepsilon (t)}}{2} + \gamma (2a + c \gamma ) \sqrt{\varepsilon (t)} \left( \frac{2 \dot{\lambda }(t)}{\lambda (t)} - \frac{{\dot{\varepsilon }}(t)}{\varepsilon (t)} \right) ^2 $$.

### Proof

We start with computing the derivative of the energy function (10). Let us denote $v(t) = \gamma \sqrt{\varepsilon (t)} \left( x(t) - x_{\varepsilon (t), \lambda (t)} \right) + \dot{x}(t) + \beta \nabla \Phi _{\lambda (t)} (x(t))$. Once again, by the classical derivation chain rule using (1) from Lemma 2 and (3) we obtain for all $t \ge t_0$

$$\begin{aligned} \dot{E}(t)&= \langle \nabla \varphi _{\varepsilon (t), \lambda (t)} (x(t)), \dot{x}(t) \rangle + \frac{{\dot{\varepsilon }}(t)}{2} \Vert x(t) \Vert ^2 + \frac{1}{2} \left( {\dot{\lambda }}(t) \varepsilon ^2(t) - \dot{\varepsilon }(t) \right) \Vert x_{\varepsilon (t), \lambda (t)} \Vert ^2 \\&\quad + \ \langle \dot{v}(t), v(t) \rangle - \frac{{\dot{\lambda }}(t)}{2} \Vert \nabla \Phi _{\lambda (t)} (x(t)) \Vert ^2. \end{aligned}$$

Our nearest goal is to obtain the upper bound for $\dot{E}$. Let us calculate for all $t \ge t_0$

$$\begin{aligned} \dot{v}(t) \ {}&= \ \frac{\gamma {\dot{\varepsilon }}(t)}{2 \sqrt{\varepsilon (t)}} \left( x(t) - x_{\varepsilon (t), \lambda (t)} \right) + \gamma \sqrt{\varepsilon (t)} \dot{x}(t) - \gamma \sqrt{\varepsilon (t)} \frac{d}{dt} x_{\varepsilon (t), \lambda (t)} \\ {}&\quad + \ddot{x}(t) + \beta \frac{d}{dt} \left( \nabla \Phi _{\lambda (t)} (x(t)) \right) \\ {}&= \ \frac{\gamma {\dot{\varepsilon }}(t)}{2 \sqrt{\varepsilon (t)}} \left( x(t) - x_{\varepsilon (t), \lambda (t)} \right) + \left( \gamma - \alpha \right) \sqrt{\varepsilon (t)} \dot{x}(t) - \gamma \sqrt{\varepsilon (t)} \frac{d}{dt} x_{\varepsilon (t), \lambda (t)} \\ {}&\quad - \nabla \Phi _{\lambda (t)} (x(t)) - \varepsilon (t) x(t) \\ {}&= \ \frac{\gamma {\dot{\varepsilon }}(t)}{2 \sqrt{\varepsilon (t)}} \left( x(t) - x_{\varepsilon (t), \lambda (t)} \right) + \left( \gamma - \alpha \right) \sqrt{\varepsilon (t)} \dot{x}(t) - \gamma \sqrt{\varepsilon (t)} \frac{d}{dt} x_{\varepsilon (t), \lambda (t)} \\ {}&\quad - \nabla \varphi _{\varepsilon (t), \lambda (t)} (x(t)), \end{aligned}$$

where above we used (1). Thus, for all $t \ge t_0$

$$\begin{aligned} \langle \dot{v}(t), v(t) \rangle&= \frac{\gamma ^2 \dot{\varepsilon }(t)}{2} \Vert x(t) - x_{\varepsilon (t), \lambda (t)} \Vert ^2 + \left( \frac{\gamma {\dot{\varepsilon }}(t)}{2 \sqrt{\varepsilon (t)}} + \gamma (\gamma - \alpha ) \varepsilon (t) \right) \\ {}&\quad \langle x(t) - x_{\varepsilon (t), \lambda (t)}, \dot{x}(t) \rangle \\ {}&\quad - \ \gamma ^2 \varepsilon (t) \left\langle \frac{d}{dt} x_{\varepsilon (t), \lambda (t)}, x(t) - x_{\varepsilon (t), \lambda (t)} \right\rangle - \gamma \sqrt{\varepsilon (t)} \\&\quad \left\langle x(t) - x_{\varepsilon (t), \lambda (t)}, \nabla \varphi _{\varepsilon (t), \lambda (t)} (x(t)) \right\rangle \\ {}&\quad + \ \left( \gamma - \alpha \right) \sqrt{\varepsilon (t)} \Vert \dot{x}(t) \Vert ^2 - \gamma \sqrt{\varepsilon (t)} \left\langle \frac{d}{dt} x_{\varepsilon (t), \lambda (t)}, \dot{x}(t) \right\rangle \\&\quad - \left\langle \nabla \varphi _{\varepsilon (t), \lambda (t)} (x(t)), \dot{x}(t) \right\rangle \\ {}&\quad + \ \frac{\gamma \beta {\dot{\varepsilon }}(t)}{2 \sqrt{\varepsilon (t)}} \left\langle x(t) - x_{\varepsilon (t), \lambda (t)}, \nabla \Phi _{\lambda (t)} (x(t)) \right\rangle \\&\quad + \beta \left( \gamma - \alpha \right) \sqrt{\varepsilon (t)} \left\langle \nabla \Phi _{\lambda (t)} (x(t)), \dot{x}(t) \right\rangle \\&\quad - \gamma \beta \sqrt{\varepsilon (t)} \left\langle \frac{d}{dt} x_{\varepsilon (t), \lambda (t)}, \nabla \Phi _{\lambda (t)} (x(t)) \right\rangle \\&\quad - \beta \left\langle \nabla \varphi _{\varepsilon (t), \lambda (t)} (x(t)), \nabla \Phi _{\lambda (t)} (x(t)) \right\rangle . \end{aligned}$$

Let us use the previous estimates to evaluate the quantity $\langle \dot{v}(t), v(t) \rangle$. Namely, by the $\varepsilon (t)$-strong convexity of $\varphi _{\varepsilon (t), \lambda (t)}$ for all $t \ge t_0$

$$\begin{aligned} \varphi _{\varepsilon (t), \lambda (t)} (x_{\varepsilon (t), \lambda (t)}) - \varphi _{\varepsilon (t), \lambda (t)} (x(t))\ge & {} \left\langle \nabla \varphi _{\varepsilon (t), \lambda (t)} (x(t)), x_{\varepsilon (t), \lambda (t)} - x(t) \right\rangle \\{} & {} + \frac{\varepsilon (t)}{2} \Vert x_{\varepsilon (t), \lambda (t)} - x(t) \Vert ^2 \end{aligned}$$

and then for all $t \ge t_0$

$$\begin{aligned}&- \gamma \sqrt{\varepsilon (t)} \left\langle x(t) - x_{\varepsilon (t), \lambda (t)}, \nabla \varphi _{\varepsilon (t), \lambda (t)} (x(t)) \right\rangle \\ {}&\quad \le - \gamma \sqrt{\varepsilon (t)} \left( \varphi _{\varepsilon (t), \lambda (t)} (x(t)) - \varphi _{\varepsilon (t), \lambda (t)} (x_{\varepsilon (t), \lambda (t)}) \right) - \frac{\gamma \varepsilon ^{\frac{3}{2}}(t)}{2} \Vert x_{\varepsilon (t), \lambda (t)} - x(t) \Vert ^2. \end{aligned}$$

Again, by the $\varepsilon (t)$-strong convexity of $\varphi _{\varepsilon (t), \lambda (t)}$ since ${\dot{\varepsilon }}(t) \ \le \ 0$ for all $t \ge t_0$

$$\begin{aligned}&\frac{\gamma \beta {\dot{\varepsilon }}(t)}{2 \sqrt{\varepsilon (t)}} \left\langle x(t) - x_{\varepsilon (t), \lambda (t)}, \nabla \Phi _{\lambda (t)} (x(t)) + \varepsilon (t) x(t) - \varepsilon (t) x(t) \right\rangle \\ {}&\quad \le \frac{\gamma \beta {\dot{\varepsilon }}(t)}{2 \sqrt{\varepsilon (t)}} \Bigg ( \varphi _{\varepsilon (t), \lambda (t)} (x(t)) - \varphi _{\varepsilon (t), \lambda (t)} (x_{\varepsilon (t), \lambda (t)}) - \frac{\varepsilon (t)}{2} \Vert x(t) - x_{\varepsilon (t), \lambda (t)} \Vert ^2 \\ {}&\qquad - \varepsilon (t) \left\langle x(t) - x_{\varepsilon (t), \lambda (t)}, x(t) \right\rangle \Bigg ). \end{aligned}$$

Furthermore,

$$\begin{aligned} - \varepsilon (t) \left\langle x(t) - x_{\varepsilon (t), \lambda (t)}, x(t) \right\rangle \ = \ -\frac{\varepsilon (t)}{2} \Big ( \Vert x(t) - x_{\varepsilon (t), \lambda (t)} \Vert ^2 + \Vert x(t) \Vert ^2 - \Vert x_{\varepsilon (t), \lambda (t)} \Vert ^2 \Big ). \end{aligned}$$

It is true that for all $a > 0$

$$\begin{aligned} - \gamma \sqrt{\varepsilon (t)} \left\langle \frac{d}{dt} x_{\varepsilon (t), \lambda (t)}, \dot{x}(t) \right\rangle \ \le \ \frac{\gamma \sqrt{\varepsilon (t)}}{2a} \Vert \dot{x}(t) \Vert ^2 + \frac{a \gamma \sqrt{\varepsilon (t)}}{2} \left\| \frac{d}{dt} x_{\varepsilon (t), \lambda (t)} \right\| ^2 \end{aligned}$$

as well as

$$\begin{aligned} - \gamma \beta \sqrt{\varepsilon (t)} \left\langle \frac{d}{dt} x_{\varepsilon (t), \lambda (t)}, \nabla \Phi _{\lambda (t)} (x(t)) \right\rangle\le & {} \frac{\gamma \beta ^2 \sqrt{\varepsilon (t)}}{2a} \Vert \nabla \Phi _{\lambda (t)} (x(t)) \Vert ^2 \\{} & {} + \frac{a \gamma \sqrt{\varepsilon (t)}}{2} \left\| \frac{d}{dt} x_{\varepsilon (t), \lambda (t)} \right\| ^2. \end{aligned}$$

In the same spirit for all $b > 0$

$$\begin{aligned} - \gamma ^2 \varepsilon (t) \left\langle \frac{d}{dt} x_{\varepsilon (t), \lambda (t)}, x(t) - x_{\varepsilon (t), \lambda (t)} \right\rangle\le & {} \frac{b \gamma \sqrt{\varepsilon (t)}}{2} \left\| \frac{d}{dt} x_{\varepsilon (t), \lambda (t)} \right\| ^2 \\{} & {} + \frac{\gamma ^3 \varepsilon ^{\frac{3}{2}}(t)}{2b} \Vert x(t) - x_{\varepsilon (t), \lambda (t)} \Vert ^2. \end{aligned}$$

Furthermore,

$$\begin{aligned}&\left( \gamma - \alpha \right) \sqrt{\varepsilon (t)} \left( \Vert \dot{x}(t) \Vert ^2 + \beta \left\langle \nabla \Phi _{\lambda (t)} (x(t)), \dot{x}(t) \right\rangle \right) \\ {}&\quad =\frac{\left( \gamma - \alpha \right) \sqrt{\varepsilon (t)}}{2} \Big ( \Vert \dot{x}(t) \Vert ^2 + \Vert \dot{x}(t) + \beta \nabla \Phi _{\lambda (t)} (x(t)) \Vert ^2 \\ {}&\qquad - \ \beta ^2 \Vert \nabla \Phi _{\lambda (t)} (x(t)) \Vert ^2 \Big ) \end{aligned}$$

and

15 $$\begin{aligned} \begin{aligned}&- \beta \left\langle \nabla \varphi _{\varepsilon (t), \lambda (t)} (x(t)), \nabla \Phi _{\lambda (t)} (x(t)) \right\rangle \\&\quad = - \frac{\beta }{2} \Big ( \Vert \nabla \varphi _{\varepsilon (t), \lambda (t)} (x(t)) \Vert ^2 + \Vert \nabla \Phi _{\lambda (t)} (x(t)) \Vert ^2 - \Vert \nabla \varphi _{\varepsilon (t), \lambda (t)} (x(t)) - \nabla \Phi _{\lambda (t)} (x(t)) \Vert ^2 \Big ) \\ {}&\quad = - \frac{\beta }{2} \Big ( \Vert \nabla \varphi _{\varepsilon (t), \lambda (t)} (x(t)) \Vert ^2 + \Vert \nabla \Phi _{\lambda (t)} (x(t)) \Vert ^2 - \varepsilon ^2(t) \Vert x(t) \Vert ^2 \Big ) \end{aligned} \end{aligned}$$

Combining all the estimates above we arrive for all $t \ge t_0$ at

$$\begin{aligned} \langle \dot{v}(t), v(t) \rangle&\le \left( \frac{\gamma \beta {\dot{\varepsilon }}(t)}{2 \sqrt{\varepsilon (t)}} - \gamma \sqrt{\varepsilon (t)} \right) \left( \varphi _{\varepsilon (t), \lambda (t)} (x(t)) - \varphi _{\varepsilon (t), \lambda (t)} (x_{\varepsilon (t), \lambda (t)}) \right) \\&\quad + \ \left( \frac{\gamma {\dot{\varepsilon }}(t)}{2 \sqrt{\varepsilon (t)}} + \gamma (\gamma - \alpha ) \varepsilon (t) \right) \langle x(t) - x_{\varepsilon (t), \lambda (t)}, \dot{x}(t) \rangle \\&\quad + \left( \frac{\gamma ^2 \dot{\varepsilon }(t)}{2} + \frac{\gamma ^3 \varepsilon ^{\frac{3}{2}}(t)}{2b} - \frac{\gamma \varepsilon ^{\frac{3}{2}}(t)}{2} - \frac{\gamma \beta \dot{\varepsilon }(t) \sqrt{\varepsilon (t)}}{2} \right) \Vert x(t) - x_{\varepsilon (t), \lambda (t)} \Vert ^2 \\&\quad + \left( \frac{\gamma }{a} + \gamma - \alpha \right) \frac{\sqrt{\varepsilon (t)}}{2} \Vert \dot{x}(t) \Vert ^2 + \frac{(\gamma - \alpha ) \sqrt{\varepsilon (t)}}{2} \Vert \dot{x}(t) + \beta \nabla \Phi _{\lambda (t)} (x(t)) \Vert ^2 \\&\quad + \ \frac{\gamma (2a + b) \sqrt{\varepsilon (t)}}{2} \left\| \frac{d}{dt} x_{\varepsilon (t), \lambda (t)} \right\| ^2 \\&\quad + \frac{1}{2} \left( \frac{\gamma \beta ^2 \sqrt{\varepsilon (t)}}{a} - \beta - \beta ^2 (\gamma - \alpha ) \sqrt{\varepsilon (t)} \right) \Vert \nabla \Phi _{\lambda (t)} (x(t)) \Vert ^2 \\&\quad - \ \left\langle \nabla \varphi _{\varepsilon (t), \lambda (t)} (x(t)), \dot{x}(t) \right\rangle + \left( \frac{\beta \varepsilon ^2(t)}{2} \right. \\&\quad \left. - \frac{\gamma \beta {\dot{\varepsilon }}(t) \sqrt{\varepsilon (t)}}{4} \right) \Vert x(t) \Vert ^2 - \frac{\beta }{2} \Vert \nabla \varphi _{\varepsilon (t), \lambda (t)} (x(t)) \Vert ^2 \\ {}&\quad + \ \frac{\gamma \beta \dot{\varepsilon }(t) \sqrt{\varepsilon (t)}}{4} \Vert x_{\varepsilon (t), \lambda (t)} \Vert ^2. \end{aligned}$$

Returning to the expression for $\dot{E}(t)$ we notice that the terms $\left\langle \nabla \varphi _{\varepsilon (t), \lambda (t)} (x(t)), \dot{x}(t) \right\rangle$ cancel each other out.

$$\begin{aligned} \dot{E}(t)&leq \left( \frac{\gamma \beta {\dot{\varepsilon }}(t)}{2 \sqrt{\varepsilon (t)}} - \gamma \sqrt{\varepsilon (t)} \right) \left( \varphi _{\varepsilon (t), \lambda (t)} (x(t)) - \varphi _{\varepsilon (t), \lambda (t)} (x_{\varepsilon (t), \lambda (t)}) \right) \\ {}&\quad + \ \left( \frac{\gamma \dot{\varepsilon }(t)}{2 \sqrt{\varepsilon (t)}} + \gamma (\gamma - \alpha ) \varepsilon (t) \right) \langle x(t) - x_{\varepsilon (t), \lambda (t)}, \dot{x}(t) \rangle \\ {}&\quad + \ \left( \frac{\gamma ^2 \dot{\varepsilon }(t)}{2} + \frac{\gamma ^3 \varepsilon ^{\frac{3}{2}}(t)}{2b} - \frac{\gamma \varepsilon ^{\frac{3}{2}}(t)}{2} - \frac{\gamma \beta \dot{\varepsilon }(t) \sqrt{\varepsilon (t)}}{2} \right) \Vert x(t) - x_{\varepsilon (t), \lambda (t)} \Vert ^2 \\ {}&\quad + \ \left( \frac{\gamma }{a} + \gamma - \alpha \right) \frac{\sqrt{\varepsilon (t)}}{2} \Vert \dot{x}(t) \Vert ^2 + \frac{(\gamma - \alpha ) \sqrt{\varepsilon (t)}}{2} \Vert \dot{x}(t) + \beta \nabla \Phi _{\lambda (t)} (x(t)) \Vert ^2 \\ {}&\quad + \ \frac{\gamma (2a + b) \sqrt{\varepsilon (t)}}{2} \left\| \frac{d}{dt} x_{\varepsilon (t), \lambda (t)} \right\| ^2 \\ {}&\quad + \ \frac{1}{2} \left( \frac{\gamma \beta ^2 \sqrt{\varepsilon (t)}}{a} - \beta - \beta ^2 (\gamma - \alpha ) \sqrt{\varepsilon (t)} - \dot{\lambda }(t) \right) \Vert \nabla \Phi _{\lambda (t)} (x(t)) \Vert ^2 \\ {}&\quad + \ \frac{1}{2} \left( {\dot{\lambda }}(t) \varepsilon ^2(t) - \dot{\varepsilon }(t) + \frac{\gamma \beta {\dot{\varepsilon }}(t) \sqrt{\varepsilon (t)}}{2} \right) \Vert x_{\varepsilon (t), \lambda (t)} \Vert ^2 \\ {}&\quad + \ \left( \frac{\beta \varepsilon ^2(t) + \dot{\varepsilon }(t)}{2} - \frac{\gamma \beta {\dot{\varepsilon }}(t) \sqrt{\varepsilon (t)}}{4} \right) \Vert x(t) \Vert ^2 \\ {}&\quad - \ \frac{\beta }{2} \Vert \nabla \varphi _{\varepsilon (t), \lambda (t)} (x(t)) \Vert ^2 \text { for all } t \ge t_0. \end{aligned}$$

Let us now consider

$$\begin{aligned} \mu (t) E(t)&= \mu (t) \left( \varphi _{\varepsilon (t), \lambda (t)} (x(t)) - \varphi _{\varepsilon (t), \lambda (t)} (x_{\varepsilon (t), \lambda (t)}) \right) \\ {}&\quad + \ \frac{\mu (t)}{2} \left\| \gamma \sqrt{\varepsilon (t)} \left( x(t) - x_{\varepsilon (t), \lambda (t)} \right) + \dot{x}(t) + \beta \nabla \Phi _{\lambda (t)} (x(t)) \right\| ^2 \\ {}&= \mu (t) \left( \varphi _{\varepsilon (t), \lambda (t)} (x(t)) - \varphi _{\varepsilon (t), \lambda (t)} (x_{\varepsilon (t), \lambda (t)}) \right) \\ {}&\quad + \frac{\gamma ^2 \mu (t) \varepsilon (t)}{2} \Vert x(t) - x_{\varepsilon (t), \lambda (t)} \Vert ^2 \\ {}&\quad + \frac{\mu (t)}{2} \Vert \dot{x}(t) + \beta \nabla \Phi _{\lambda (t)} (x(t)) \Vert ^2\\ {}&\quad + \gamma \mu (t) \sqrt{\varepsilon (t)} \left\langle x(t) - x_{\varepsilon (t), \lambda (t)}, \dot{x}(t) + \beta \nabla \Phi _{\lambda (t)} (x(t)) \right\rangle \\ {}&\le \mu (t) \left( \varphi _{\varepsilon (t), \lambda (t)} (x(t)) - \varphi _{\varepsilon (t), \lambda (t)} (x_{\varepsilon (t), \lambda (t)}) \right) \\ {}&\quad + \gamma ^2 \mu (t) \varepsilon (t) \Vert x(t) - x_{\varepsilon (t), \lambda (t)} \Vert ^2 \\ {}&\quad + \frac{\mu (t)}{2} \Vert \dot{x}(t) + \beta \nabla \Phi _{\lambda (t)} (x(t)) \Vert ^2 + \gamma \mu (t) \sqrt{\varepsilon (t)} \left\langle x(t) - x_{\varepsilon (t), \lambda (t)}, \dot{x}(t) \right\rangle \\&\quad + \frac{\beta ^2 \mu (t)}{2} \Vert \nabla \Phi _{\lambda (t)} (x(t)) \Vert ^2, \end{aligned}$$

since

$$\begin{aligned}&\gamma \beta \mu (t) \sqrt{\varepsilon (t)} \left\langle x(t) - x_{\varepsilon (t), \lambda (t)}, \nabla \Phi _{\lambda (t)} (x(t)) \right\rangle \ \le \ \gamma \beta \mu (t) \sqrt{\varepsilon (t)} \Vert x(t) \\ {}&\quad - x_{\varepsilon (t), \lambda (t)} \Vert \Vert \nabla \Phi _{\lambda (t)} (x(t)) \Vert \\ \le \ {}&\frac{\gamma ^2 \mu (t) \varepsilon (t)}{2} \Vert x(t) - x_{\varepsilon (t), \lambda (t)} \Vert ^2 + \frac{\beta ^2 \mu (t)}{2} \Vert \nabla \Phi _{\lambda (t)} (x(t)) \Vert ^2. \end{aligned}$$

Therefore, using $\mu (t) = \left( \alpha - \gamma \right) \sqrt{\varepsilon (t)} - \frac{{\dot{\varepsilon }}(t)}{2 \varepsilon (t)}$ (the terms with $\langle x(t) - x_{\varepsilon (t), \lambda (t)}, \dot{x}(t) \rangle$ disappear), we obtain for all $t \ge t_0$

$$\begin{aligned}&\dot{E}(t) + \mu (t) E(t) \ \le \ \left( \frac{\gamma \beta \dot{\varepsilon }(t)}{2 \sqrt{\varepsilon (t)}} + (\alpha - 2 \gamma ) \sqrt{\varepsilon (t)} - \frac{{\dot{\varepsilon }}(t)}{2 \varepsilon (t)} \right) \\ {}&\quad \left( \varphi _{\varepsilon (t), \lambda (t)} (x(t)) - \varphi _{\varepsilon (t), \lambda (t)} (x_{\varepsilon (t), \lambda (t)}) \right) \\ {}&\quad + \ \left( \gamma ^2 (\alpha - \gamma ) \varepsilon ^{\frac{3}{2}}(t) + \frac{\gamma ^3 \varepsilon ^{\frac{3}{2}}(t)}{2b} - \frac{\gamma \varepsilon ^{\frac{3}{2}}(t)}{2} - \frac{\gamma \beta \dot{\varepsilon }(t) \sqrt{\varepsilon (t)}}{2} \right) \Vert x(t) - x_{\varepsilon (t), \lambda (t)} \Vert ^2 \\ {}&\quad + \ \left( \frac{\gamma }{a} + \gamma - \alpha \right) \frac{\sqrt{\varepsilon (t)}}{2} \Vert \dot{x}(t) \Vert ^2 - \frac{{\dot{\varepsilon }}(t) }{4 \varepsilon (t)} \Vert \dot{x}(t) + \beta \nabla \Phi _{\lambda (t)} (x(t)) \Vert ^2 \\ {}&\quad + \ \frac{\gamma (2a + b) \sqrt{\varepsilon (t)}}{2} \left\| \frac{d}{dt} x_{\varepsilon (t), \lambda (t)} \right\| ^2 \\ {}&\quad + \ \frac{1}{2} \left( \frac{\gamma \beta ^2 \sqrt{\varepsilon (t)}}{a} - \beta + 2 \beta ^2 (\alpha - \gamma ) \sqrt{\varepsilon (t)} - \frac{\beta ^2 {\dot{\varepsilon }}(t)}{2 \varepsilon (t)} - \dot{\lambda }(t) \right) \Vert \nabla \Phi _{\lambda (t)} (x(t)) \Vert ^2 \\ {}&\quad + \ \frac{1}{2} \left( {\dot{\lambda }}(t) \varepsilon ^2(t) - \dot{\varepsilon }(t) + \frac{\gamma \beta {\dot{\varepsilon }}(t) \sqrt{\varepsilon (t)}}{2} \right) \Vert x_{\varepsilon (t), \lambda (t)} \Vert ^2 \\ {}&\quad + \left( \frac{\beta \varepsilon ^2(t) + {\dot{\varepsilon }}(t)}{2} - \frac{\gamma \beta {\dot{\varepsilon }}(t) \sqrt{\varepsilon (t)}}{4} \right) \Vert x(t) \Vert ^2 \\ {}&\quad - \ \frac{\beta }{2} \Vert \nabla \varphi _{\varepsilon (t), \lambda (t)} (x(t)) \Vert ^2. \end{aligned}$$

Further we have (${\dot{\varepsilon }}(t) \le 0$ for all $t \ge t_0$)

$$\begin{aligned} - \frac{{\dot{\varepsilon }}(t) }{4 \varepsilon (t)} \Vert \dot{x}(t) + \beta \nabla \Phi _{\lambda (t)} (x(t)) \Vert ^2 \ \le \ - \frac{\dot{\varepsilon }(t) }{2 \varepsilon (t)} \Vert \dot{x}(t) \Vert ^2 - \frac{\beta ^2 {\dot{\varepsilon }}(t) }{2 \varepsilon (t)} \Vert \nabla \Phi _{\lambda (t)} (x(t)) \Vert ^2. \end{aligned}$$

As we have established earlier by Lemma 2 item 2 and (8)

$$\begin{aligned} \left\| \frac{d}{dt} x_{\varepsilon (t), \lambda (t)} \right\| ^2 \ \le \ \left( \frac{2 {\dot{\lambda }}(t)}{\lambda (t)} - \frac{\dot{\varepsilon }(t)}{\varepsilon (t)} \right) ^2 \Vert x_{\varepsilon (t), \lambda (t)} \Vert ^2 \ \le \ \left( \frac{2 \dot{\lambda }(t)}{\lambda (t)} - \frac{{\dot{\varepsilon }}(t)}{\varepsilon (t)} \right) ^2 \Vert x^* \Vert ^2, \end{aligned}$$

and since there exists $t_1 \ge t_0$ such that $\left( \sqrt{\varepsilon (t)} \rightarrow 0, \text { as } t \rightarrow +\infty \right)$

$$\begin{aligned} {\dot{\lambda }}(t) \varepsilon ^2(t) + \left( \frac{\gamma \beta \sqrt{\varepsilon (t)}}{2} - 1 \right) {\dot{\varepsilon }}(t) \ \ge \ 0 \text { for all } t \ge t_1, \end{aligned}$$

we deduce for all $t \ge t_1$

$$\begin{aligned}{} & {} \frac{1}{2} \left( {\dot{\lambda }}(t) \varepsilon ^2(t) - \dot{\varepsilon }(t) + \frac{\gamma \beta {\dot{\varepsilon }}(t) \sqrt{\varepsilon (t)}}{2} \right) \Vert x_{\varepsilon (t), \lambda (t)} \Vert ^2 \ \\ {}{} & {} \quad \le \ \frac{1}{2} \left( {\dot{\lambda }}(t) \varepsilon ^2(t) - {\dot{\varepsilon }}(t) + \frac{\gamma \beta {\dot{\varepsilon }}(t) \sqrt{\varepsilon (t)}}{2} \right) \Vert x^* \Vert ^2. \end{aligned}$$

Choosing $b = c \gamma$ with $c > 0$ we obtain for all $t \ge t_1$

$$\begin{aligned}&\dot{E}(t) + \mu (t) E(t) \ \le \ \left( \frac{\gamma \beta \dot{\varepsilon }(t)}{2 \sqrt{\varepsilon (t)}} + (\alpha - 2 \gamma ) \sqrt{\varepsilon (t)} - \frac{{\dot{\varepsilon }}(t)}{2 \varepsilon (t)} \right) \\ {}&\quad \left( \varphi _{\varepsilon (t), \lambda (t)} (x(t)) - \varphi _{\varepsilon (t), \lambda (t)} (x_{\varepsilon (t), \lambda (t)}) \right) \\&\quad + \ \left( \gamma ^2 (\alpha - \gamma ) \varepsilon ^{\frac{3}{2}}(t) + \frac{\gamma ^2 \varepsilon ^{\frac{3}{2}}(t)}{2c} - \frac{\gamma \varepsilon ^{\frac{3}{2}}(t)}{2} - \frac{\gamma \beta \dot{\varepsilon }(t) \sqrt{\varepsilon (t)}}{2} \right) \Vert x(t) - x_{\varepsilon (t), \lambda (t)} \Vert ^2 \\&\quad + \ \left( \frac{\gamma }{a} + \gamma - \alpha - \frac{\dot{\varepsilon }(t)}{\varepsilon ^{\frac{3}{2}}(t)} \right) \frac{\sqrt{\varepsilon (t)}}{2} \Vert \dot{x}(t) \Vert ^2 \\&\quad + \ \frac{1}{2} \left( \frac{\gamma \beta ^2 \sqrt{\varepsilon (t)}}{a} - \beta + 2 \beta ^2 (\alpha - \gamma ) \sqrt{\varepsilon (t)} - \frac{3 \beta ^2 {\dot{\varepsilon }}(t)}{2 \varepsilon (t)} - {\dot{\lambda }}(t) \right) \Vert \nabla \Phi _{\lambda (t)} (x(t)) \Vert ^2 \\&\quad + \ \left( \frac{\beta \varepsilon ^2(t) + {\dot{\varepsilon }}(t)}{2} - \frac{\gamma \beta {\dot{\varepsilon }}(t) \sqrt{\varepsilon (t)}}{4} \right) \Vert x(t) \Vert ^2 \\&\quad + \ \left( \frac{1}{2} \left( {\dot{\lambda }}(t) \varepsilon ^2(t) - {\dot{\varepsilon }}(t) + \frac{\gamma \beta {\dot{\varepsilon }}(t) \sqrt{\varepsilon (t)}}{2} \right) \right. \\ {}&\quad \left. + \frac{\gamma (2a + c \gamma ) \sqrt{\varepsilon (t)}}{2} \left( \frac{2 \dot{\lambda }(t)}{\lambda (t)} - \frac{{\dot{\varepsilon }}(t)}{\varepsilon (t)} \right) ^2 \right) \Vert x^* \Vert ^2 \\&\quad - \frac{\beta }{2} \Vert \nabla \varphi _{\varepsilon (t), \lambda (t)} (x(t)) \Vert ^2. \end{aligned}$$

Let us investigate the signs of the terms in the inequality above when *t* is large enough to satisfy what we assumed before (11)–(14). First of all,

$$\begin{aligned} (\alpha - 2 \gamma ) \sqrt{\varepsilon (t)} - \frac{\dot{\varepsilon }(t)}{2 \varepsilon (t)} + \frac{\gamma \beta \dot{\varepsilon }(t)}{2 \sqrt{\varepsilon (t)}} \ = \ \left( \frac{d}{dt} \left( \frac{1}{\sqrt{\varepsilon (t)}} \right) + \alpha - 2\gamma + \frac{\gamma \beta {\dot{\varepsilon }}(t)}{2 \varepsilon (t)} \right) \sqrt{\varepsilon (t)} \ \le \ 0 \end{aligned}$$

due to (11). Secondly,

$$\begin{aligned}&\gamma ^2 (\alpha - \gamma ) \varepsilon ^{\frac{3}{2}}(t) + \frac{\gamma ^2 \varepsilon ^{\frac{3}{2}}(t)}{2c} - \frac{\gamma \varepsilon ^{\frac{3}{2}}(t)}{2} - \frac{\gamma \beta \dot{\varepsilon }(t) \sqrt{\varepsilon (t)}}{2} \\ {}&\quad = \ \frac{\gamma \varepsilon ^{\frac{3}{2}}(t)}{2} \left( 2 \gamma (\alpha - \gamma ) + \frac{\gamma }{c} - 1 \right) - \frac{\gamma \beta \dot{\varepsilon }(t) \sqrt{\varepsilon (t)}}{2} \\ {}&\quad = x\frac{\gamma \sqrt{\varepsilon (t)}}{2} \left( \left( 2 \gamma (\alpha - \gamma ) + \frac{\gamma }{c} - 1 \right) \varepsilon (t) - \beta \dot{\varepsilon }(t) \right) \ \le \ 0 \end{aligned}$$

due to (12). Next we have

$$\begin{aligned} \frac{\gamma }{a} + \gamma - \alpha - \frac{\dot{\varepsilon }(t)}{\varepsilon ^{\frac{3}{2}}(t)} \ = \ \frac{d}{dt} \left( \frac{1}{\sqrt{\varepsilon (t)}} \right) + \gamma \frac{a + 1}{a} - \alpha \ \le \ 0 \end{aligned}$$

due to (11). Then,

$$\begin{aligned} \frac{\gamma \beta ^2 \sqrt{\varepsilon (t)}}{a} - \beta + 2 \beta ^2 (\alpha - \gamma ) \sqrt{\varepsilon (t)} - \frac{3 \beta ^2 \dot{\varepsilon }(t)}{2 \varepsilon (t)} - {\dot{\lambda }}(t) \ \le \ 0 \end{aligned}$$

due to (14). Finally,

$$\begin{aligned} \frac{\beta \varepsilon ^2(t) + {\dot{\varepsilon }}(t)}{2} - \frac{\gamma \beta {\dot{\varepsilon }}(t) \sqrt{\varepsilon (t)}}{4} \ \le \ 0 \end{aligned}$$

due to (13), since

$$\begin{aligned} 2 \beta \varepsilon ^2(t) + \left( 2 - \gamma \beta \sqrt{\varepsilon (t)} \right) {\dot{\varepsilon }}(t) \ \le \ 0. \end{aligned}$$

So, at the end we deduce for all $t \ge t_1$

16 $$\begin{aligned} \begin{aligned} \dot{E}(t) + \mu (t) E(t) \ \le \ {}&\frac{1}{2} \left( {\dot{\lambda }}(t) \varepsilon ^2(t) - {\dot{\varepsilon }}(t) + \frac{\gamma \beta \dot{\varepsilon }(t) \sqrt{\varepsilon (t)}}{2} \right. \\ {}&+\left. \gamma (2a + c \gamma ) \sqrt{\varepsilon (t)} \left( \frac{2 {\dot{\lambda }}(t)}{\lambda (t)} - \frac{{\dot{\varepsilon }}(t)}{\varepsilon (t)} \right) ^2 \right) \Vert x^* \Vert ^2 \\ {}&- \ \frac{\beta }{2} \Vert \nabla \varphi _{\varepsilon (t), \lambda (t)} (x(t)) \Vert ^2 \ = \ \frac{g(t) \Vert x^* \Vert ^2}{2} - \frac{\beta }{2} \Vert \nabla \varphi _{\varepsilon (t), \lambda (t)} (x(t)) \Vert ^2. \end{aligned} \end{aligned}$$

Integrating (16) from $t_1$ to *t* we obtain

$$\begin{aligned} E(t) - E(t_1) + \int _{t_1}^t \mu (s) E(s) ds + \frac{\beta }{2} \int _{t_1}^t \Vert \nabla \varphi _{\varepsilon (s)} (x(s)) \Vert ^2 ds \ \le \ \frac{\Vert x^* \Vert ^2}{2} \int _{t_1}^t g(s) ds \end{aligned}$$

or, neglecting the positive terms,

$$\begin{aligned} \frac{\beta }{2} \int _{t_1}^t \Vert \nabla \varphi _{\varepsilon (s)} (x(s)) \Vert ^2 ds \ \le \ E(t_1) + \frac{\Vert x^* \Vert ^2}{2} \int _{t_1}^t g(s) ds. \end{aligned}$$

From (16) we also obtain for all $t \ge t_1$

$$\begin{aligned} \dot{E}(t) + \mu (t) E(t) \ \le \ \frac{g(t) \Vert x^* \Vert ^2}{2}. \end{aligned}$$

Multiplying this with $\Gamma (t) = \exp \left( \int _{t_1}^t \mu (s) ds \right)$ and integrating again on $[t_1, t]$ we deduce

$$\begin{aligned} E(t) \ \le \ \frac{\Vert x^* \Vert ^2}{2 \Gamma (t)} \int _{t_1}^t \Gamma (s) g(s) ds + \frac{\Gamma (t_1) E(t_1)}{\Gamma (t)}. \end{aligned}$$

$$\square $$

Now that we have an estimate for the energy function as well, we are able to derive, under which conditions the quantities of the right-hand side of the estimates of the Theorem 5 do converge to zero, as $t \rightarrow +\infty$. This result is given by the next theorem.

### Theorem 7

Let $x: [t_0, +\infty ) \longrightarrow H$ be a solution of (1). Assume that (7) holds and suppose that there exist $a, c > 0$ such that for *t* large enough assumptions (11) - (14) hold. Suppose additionally that

17 $$\begin{aligned} \begin{aligned}&\lim _{t \rightarrow +\infty } \sqrt{\varepsilon (t)} \exp \left( (\alpha - \gamma ) \int _{t_1}^t \sqrt{\varepsilon (s)} ds \right) \ = \ +\infty , \\ {}&\lim _{t \rightarrow +\infty } \frac{\dot{\varepsilon }(t)}{\varepsilon ^\frac{3}{2}(t)} \ = \ 0, \\ {}&\lim _{t \rightarrow +\infty } {\dot{\lambda }}(t) \sqrt{\varepsilon (t)} \ = \ 0 \text { and } \\ {}&\lim _{t \rightarrow +\infty } \frac{{\dot{\lambda }}(t)}{\lambda (t) \sqrt{\varepsilon (t)}} \ = \ 0. \end{aligned} \end{aligned}$$

Then

$$\begin{aligned} \lim _{t \rightarrow +\infty } E(t) \ = \ \lim _{t \rightarrow +\infty } \frac{E(t)}{\varepsilon (t)} \ = \ \lim _{t \rightarrow +\infty } \frac{E(t)}{\lambda (t)} \ = \ 0. \end{aligned}$$

The proof of this theorem reader may find in the Appendix. As we can see, the results of Theorem 7 together with (7) and $\lim _{t \rightarrow +\infty } \varepsilon (t) = 0$ guarantee the convergence to zero, as $t \rightarrow +\infty$, of the quantities in Theorem 5.

## Polynomial choice of parameters

In this section we would like to specify the form of the functions $\lambda$ and $\varepsilon$, namely, taking $\lambda (t) = t^l$ and $\varepsilon (t) = \frac{1}{t^d}$, $l \ge 0$ and $d > 0$, and show that the main results still hold in this case. First of all, equation (1) becomes

18 $$\begin{aligned} \ddot{x}(t) + \frac{\alpha }{t^{\frac{d}{2}}} \dot{x}(t) + \beta \frac{d}{dt} \left( \nabla \Phi _{t^l} (x(t)) \right) + \nabla \Phi _{t^l} (x(t)) + \frac{1}{t^d} x(t) = 0 \text { for } t \ge t_0. \end{aligned}$$

The second step would be to show that the main result of Theorem 6 is valid and obtain the precise rates of convergence for the function values and trajectories using Theorem 5. In order to do so let us formulate the next theorem.

### Theorem 8

Let $x: [t_0, +\infty ) \longrightarrow H$ be a solution of (18). Assume that $1 \le d < 2$ and $0 \le l < d$. Then for *t* large enough

$$\begin{aligned}{} & {} \Phi _{\lambda (t)} (x(t)) - \Phi ^* \ \le \ \frac{1}{t^d},\\{} & {} \Phi \left( \mathop {\textrm{prox}}\limits \nolimits _{\lambda (t) \Phi }(x(t)) \right) - \Phi ^* \ \le \ \frac{1}{t^d},\\{} & {} \Vert \mathop {\textrm{prox}}\limits \nolimits _{\lambda (t) \Phi } (x(t)) - x(t) \Vert ^2 \ \le \ \frac{1}{t^{\frac{d}{2} + 1 - l}} \end{aligned}$$

and

$$\begin{aligned} \Vert x(t) - x_{\varepsilon (t), \lambda (t)} \Vert ^2 \ \le \ \frac{1}{t^{1-\frac{d}{2}}}. \end{aligned}$$

If $d = 2$ and $0 \le l < 2$ we deduce the following estimates for *t* large enough.

If $$0< \alpha < 2$$, then

$$\begin{aligned}{} & {} \Phi _{\lambda (t)} (x(t)) - \Phi ^* \ \le \ \frac{1}{t^{\frac{\alpha }{2} + 1}},\\{} & {} \Phi \left( \mathop {\textrm{prox}}\limits \nolimits _{\lambda (t) \Phi }(x(t)) \right) - \Phi ^* \ \le \ \frac{1}{t^{\frac{\alpha }{2} + 1}} \end{aligned}$$

and

$$\begin{aligned} \Vert \mathop {\textrm{prox}}\limits \nolimits _{\lambda (t) \Phi } (x(t)) - x(t) \Vert ^2 \ \le \ \frac{1}{t^{\frac{\alpha }{2} - l + 1}}. \end{aligned}$$

If $$\alpha \ge 2$$, then

$$\begin{aligned}{} & {} \Phi _{\lambda (t)} (x(t)) - \Phi ^* \ \le \ \frac{1}{t^2},\\{} & {} \Phi \left( \mathop {\textrm{prox}}\limits \nolimits _{\lambda (t) \Phi }(x(t)) \right) - \Phi ^* \ \le \ \frac{1}{t^2} \end{aligned}$$

and

$$\begin{aligned} \Vert \mathop {\textrm{prox}}\limits \nolimits _{\lambda (t) \Phi } (x(t)) - x(t) \Vert ^2 \ \le \ \frac{1}{t^{2-l}}. \end{aligned}$$

### Proof

It is easy to check (see Appendix) that the choice above of *d* and *l* satisfies conditions (7) and (11)–(14). Therefore the results of Theorem 6 are valid in this case. Namely, in Theorem 6, we have obtained

$$\begin{aligned} E(t) \ \le \ \frac{\Vert x^* \Vert ^2}{2 \Gamma (t)} \int _{t_1}^t \Gamma (s) g(s) ds + \frac{\Gamma (t_1) E(t_1)}{\Gamma (t)}, \end{aligned}$$

where $g(t) = {\dot{\lambda }}(t) \varepsilon ^2(t) - {\dot{\varepsilon }}(t) + \frac{\gamma \beta {\dot{\varepsilon }}(t) \sqrt{\varepsilon (t)}}{2} + \gamma (2a + c \gamma ) \sqrt{\varepsilon (t)} \left( \frac{2 \dot{\lambda }(t)}{\lambda (t)} - \frac{{\dot{\varepsilon }}(t)}{\varepsilon (t)} \right) ^2$, $\Gamma (t) = \exp \left( \int _{t_1}^t \mu (s) ds \right)$ and $\mu (t) = \left( \alpha - \gamma \right) \sqrt{\varepsilon (t)} - \frac{{\dot{\varepsilon }}(t)}{2 \varepsilon (t)}$. Now, our goal is to deduce the actual rates of convergence of the function values and trajectories. The proof will be divided into several sections for the convenience of the reader.

## The functions $\mu$**and**$\Gamma$

Let us consider the case when $1 \le d < 2$. The case when $d = 2$ will be treated separately. The function $\mu$ thus writes as follows $\mu (t) \ = \ \frac{\alpha - \gamma }{t^{\frac{d}{2}}} + \frac{d}{2t}$. Then,

$$\begin{aligned} \Gamma (t) \ {}&= \ \exp \left( \int _{t_1}^t \left[ \frac{\alpha - \gamma }{s^{\frac{d}{2}}} + \frac{d}{2s} \right] ds \right) \ = \ \left( \frac{t}{t_1} \right) ^\frac{d}{2} \exp \left( \int _{t_1}^t \frac{\alpha - \gamma }{s^{\frac{d}{2}}} ds \right) \\&= \ \left( \frac{t}{t_1} \right) ^\frac{d}{2} \exp \left( \frac{\alpha - \gamma }{1 - \frac{d}{2}} \left[ t^{1 - \frac{d}{2}} - t_1^{1 - \frac{d}{2}} \right] \right) \ = \ C t^\frac{d}{2} \exp \left( \frac{\alpha - \gamma }{1 - \frac{d}{2}} t^{1 - \frac{d}{2}} \right) , \end{aligned}$$

where $C = \left( t_1^\frac{d}{2} \exp \left[ \frac{\alpha - \gamma }{1 - \frac{d}{2}} t_1^{1 - \frac{d}{2}} \right] \right) ^{-1}$. So, $\frac{\Gamma (t_1) E(t_1)}{\Gamma (t)}$ goes to zero exponentially, as time goes to infinity due to $1 \le d < 2$.

## The function *g*

First notice that

$$\begin{aligned} g(t) \ {}&= \ \frac{l t^{l-1}}{t^{2d}} + \frac{d}{t^{d+1}} - \frac{\gamma \beta d}{2 t^{d + 1 + \frac{d}{2}}} + \frac{\gamma (2a + c \gamma )}{t^\frac{d}{2}} \left( \frac{2l}{t} + \frac{d}{t} \right) ^2 \\ {}&= \ l t^{l - 1 - 2d} + \frac{d}{t^{d+1}} - \frac{\gamma \beta d}{2 t^{d + 1 + \frac{d}{2}}} + \frac{\gamma (2a + c \gamma ) (2l + d)^2}{t^{\frac{d}{2} + 2}} \\ {}&= \ \frac{l}{t^{2d-l+1}} + \frac{d}{t^{d+1}} - \frac{\gamma \beta d}{2 t^{d + 1 + \frac{d}{2}}} + \frac{C_1}{t^{\frac{d}{2} + 2}}, \end{aligned}$$

where $$C_1 = \gamma (2a + c \gamma ) (2l + d)^2$$. Then,

$$\begin{aligned} \Gamma (t) g(t) \ = \ C \left( \frac{l}{t^{\frac{3d}{2} + 1 - l}} + \frac{d}{t^{\frac{d}{2}+1}} - \frac{\gamma \beta d}{2 t^{d+1}} + \frac{C_1}{t^2} \right) \exp \left( \frac{\alpha - \gamma }{1 - \frac{d}{2}} t^{1 - \frac{d}{2}} \right) . \end{aligned}$$

Let us notice that the behaviour of $\frac{l}{t^{\frac{3d}{2} + 1 - l}} + \frac{d}{t^{\frac{d}{2}+1}} - \frac{\gamma \beta d}{2 t^{d+1}} + \frac{C_1}{t^2}$ is dictated by the term $\frac{1}{t^{\frac{d}{2}+1}}$, as $t \rightarrow +\infty$, since $1 \le d < 2$ and $0 \le l < d$.

## Integrating the product $\Gamma g$

The technique, which will be used in this section, is inspired by [3]. First of all, notice that for some $\delta > 0$

$$\begin{aligned} \frac{d}{dt} \left( \frac{\exp \left( \frac{\alpha - \gamma }{1 - \frac{d}{2}} t^{1 - \frac{d}{2}} \right) }{\delta t} \right) \ = \ \left( -\frac{1}{\delta t^2} + \frac{\alpha - \gamma }{\delta t^{\frac{d}{2}+1}} \right) \exp \left( \frac{\alpha - \gamma }{1 - \frac{d}{2}} t^{1 - \frac{d}{2}} \right) . \end{aligned}$$

Secondly, there exists such $\delta$ that starting from some $t_2 \ge t_1$ it holds that

$$\begin{aligned} \frac{l}{t^{\frac{3d}{2} + 1 - l}} + \frac{d}{t^{\frac{d}{2}+1}} - \frac{\gamma \beta d}{2 t^{d+1}} + \frac{C_1}{t^2} \ \le \ -\frac{1}{\delta t^2} + \frac{\alpha - \gamma }{\delta t^{\frac{d}{2}+1}}. \end{aligned}$$

Thus,

$$\begin{aligned}&C \int _{t_2}^t \left( \frac{l}{s^{\frac{3d}{2} + 1 - l}} + \frac{d}{s^{\frac{d}{2}+1}} - \frac{\gamma \beta d}{2 s^{d+1}} + \frac{C_1}{s^2} \right) \exp \left( \frac{\alpha - \gamma }{1 - \frac{d}{2}} s^{1 - \frac{d}{2}} \right) ds \\ {}&\quad \le C \int _{t_2}^t \left( -\frac{1}{\delta s^2} + \frac{\alpha - \gamma }{\delta s^{\frac{d}{2}+1}} \right) \exp \left( \frac{\alpha - \gamma }{1 - \frac{d}{2}} s^{1 - \frac{d}{2}} \right) ds \\ {}&\quad = C \int _{t_2}^t \frac{d}{ds} \left( \frac{\exp \left( \frac{\alpha - \gamma }{1 - \frac{d}{2}} s^{1 - \frac{d}{2}} \right) }{\delta s} \right) ds \ = \ C \left( \frac{\exp \left( \frac{\alpha - \gamma }{1 - \frac{d}{2}} t^{1 - \frac{d}{2}} \right) }{\delta t} - \frac{\exp \left( \frac{\alpha - \gamma }{1 - \frac{d}{2}} t_2^{1 - \frac{d}{2}} \right) }{\delta t_2} \right) \\ {}&\quad = C \frac{\exp \left( \frac{\alpha - \gamma }{1 - \frac{d}{2}} t^{1 - \frac{d}{2}} \right) }{\delta t} - C_2, \end{aligned}$$

where $$C_2 = C \frac{\exp \left( \frac{\alpha - \gamma }{1 - \frac{d}{2}} t_2^{1 - \frac{d}{2}} \right) }{\delta t_2}$$.

## Finalizing the estimates

Let us return to

$$\begin{aligned} E(t)&\le \frac{\Vert x^* \Vert ^2}{2 \Gamma (t)} \int _{t_1}^t \Gamma (s) g(s) ds + \frac{\Gamma (t_1) E(t_1)}{\Gamma (t)} \ = \ \frac{\Vert x^* \Vert ^2}{2 \Gamma (t)} \int _{t_1}^{t_2} \Gamma (s) g(s) ds \\&\quad + \frac{\Vert x^* \Vert ^2}{2 \Gamma (t)} \int _{t_2}^t \Gamma (s) g(s) ds \\ {}&\quad + \frac{\Gamma (t_1) E(t_1)}{\Gamma (t)} \ \le \ \frac{\Vert x^* \Vert ^2}{2} \left( \frac{1}{\Gamma (t)} \int _{t_1}^{t_2} \Gamma (s) g(s) ds - \frac{C_2}{\Gamma (t)} + C \frac{\exp \left( \frac{\alpha - \gamma }{1 - \frac{d}{2}} t^{1 - \frac{d}{2}} \right) }{\delta t \Gamma (t)} \right) \\&\quad + \frac{\Gamma (t_1) E(t_1)}{\Gamma (t)}. \end{aligned}$$

This expression converges to zero at a speed of the slowest decaying term (all the other decay exponentially):

$$\begin{aligned} C \frac{\exp \left( \frac{\alpha - \gamma }{1 - \frac{d}{2}} t^{1 - \frac{d}{2}} \right) }{\delta t \Gamma (t)} \ = \ C \frac{\exp \left( \frac{\alpha - \gamma }{1 - \frac{d}{2}} t^{1 - \frac{d}{2}} \right) }{\delta C t^{\frac{d}{2}+1} \exp \left( \frac{\alpha - \gamma }{1 - \frac{d}{2}} t^{1 - \frac{d}{2}} \right) } \ = \ \frac{1}{\delta t^{\frac{d}{2}+1}}. \end{aligned}$$

Thus, there exists a constant $C_3 > 0$ such that for all $t \ge t_2$

$$\begin{aligned} E(t) \ \le \ \frac{C_3}{t^{\frac{d}{2}+1}}. \end{aligned}$$

## The rates themselves

Now we can deduce the actual rates for the quantities in Theorem 5. For all $t \ge t_2$

$$\begin{aligned}{} & {} \Phi _{\lambda (t)} (x(t)) - \Phi ^* \ \le \ \frac{C_3}{t^{\frac{d}{2}+1}} + \frac{1}{2t^d} \Vert x^* \Vert ^2,\\{} & {} \Phi \left( \mathop {\textrm{prox}}\limits \nolimits _{\lambda (t) \Phi }(x(t)) \right) - \Phi ^* \ \le \ \frac{C_3}{t^{\frac{d}{2}+1}} + \frac{1}{2t^d} \Vert x^* \Vert ^2,\\{} & {} \Vert \mathop {\textrm{prox}}\limits \nolimits _{\lambda (t) \Phi } (x(t)) - x(t) \Vert ^2 \ \le \ 2 C_3 t^{l - \frac{d}{2} - 1} + t^{l-d} \Vert x^* \Vert ^2 \end{aligned}$$

and

$$\begin{aligned} \Vert x(t) - x_{\varepsilon (t), \lambda (t)} \Vert ^2 \ \le \ \frac{C_3}{t^{1-\frac{d}{2}}}. \end{aligned}$$

Finally, there exist constants $C_4, C_5 > 0$ such that for all $t \ge t_2$

$$\begin{aligned}{} & {} \Phi _{\lambda (t)} (x(t)) - \Phi ^* \ \le \ \frac{C_4}{t^d},\\{} & {} \Phi \left( \mathop {\textrm{prox}}\limits \nolimits _{\lambda (t) \Phi }(x(t)) \right) - \Phi ^* \ \le \ \frac{C_4}{t^d},\\{} & {} \Vert \mathop {\textrm{prox}}\limits \nolimits _{\lambda (t) \Phi } (x(t)) - x(t) \Vert ^2 \ \le \ \frac{C_5}{t^{\frac{d}{2} + 1 - l}} \end{aligned}$$

and

$$\begin{aligned} \Vert x(t) - x_{\varepsilon (t), \lambda (t)} \Vert ^2 \ \le \ \frac{C_3}{t^{1-\frac{d}{2}}}. \end{aligned}$$

## The rates of convergence of the function values in case $d=2$

This particular case is of a great interest, as it is in a way a bordering case, when one cannot show the strong convergence of the trajectories, but still can show the fast convergence of the values. In this case the functions $\mu$ and $\Gamma$ are

$$\begin{aligned} \mu (t) \ = \ \frac{\alpha - \gamma + 1}{t} \end{aligned}$$

and

$$\begin{aligned} \Gamma (t) \ = \ \exp \left( \int _{t_1}^t \frac{\alpha - \gamma + 1}{s} ds \right) \ = \ \left( \frac{t}{t_1} \right) ^{\alpha - \gamma + 1} \ = \ C t^{\alpha - \gamma + 1}, \end{aligned}$$

where $C = \frac{1}{t_1^{\alpha - \gamma + 1}}$. The function *g* is

$$\begin{aligned} g(t) \ = \ \frac{l}{t^{5-l}} + \frac{2}{t^3} - \frac{\gamma \beta }{t^4} + \frac{C_1}{t^3} \ = \ \frac{l}{t^{5-l}} + \frac{2 + C_1}{t^3} - \frac{\gamma \beta }{t^4}, \end{aligned}$$

where $$C_1 = 4 \gamma (2a + c \gamma ) (l + 1)^2$$. Thus,

$$\begin{aligned} \Gamma (t) g(t) \ = \ C \left( l t^{\alpha - \gamma + l - 4} + \left( 2 + C_1 \right) t^{\alpha - \gamma - 2} - \gamma \beta t^{\alpha - \gamma - 3} \right) . \end{aligned}$$

So,

$$\begin{aligned}&C \int _{t_1}^t \left( l s^{\alpha - \gamma + l - 4} + \left( 2 + C_1 \right) s^{\alpha - \gamma - 2} - \gamma \beta s^{\alpha - \gamma - 3} \right) ds \\ {}&\quad = C \left( \frac{l s^{\alpha - \gamma + l - 3}}{\alpha - \gamma + l - 3} + \frac{\left( 2 + C_1 \right) s^{\alpha - \gamma - 1}}{\alpha - \gamma - 1} - \frac{\gamma \beta s^{\alpha - \gamma - 2}}{\alpha - \gamma - 2} \right) \Bigg |_{t_1}^t \\ {}&\quad = C \left( \frac{l t^{\alpha - \gamma + l - 3}}{\alpha - \gamma + l - 3} + \frac{\left( 2 + C_1 \right) t^{\alpha - \gamma - 1}}{\alpha - \gamma - 1} - \frac{\gamma \beta t^{\alpha - \gamma - 2}}{\alpha - \gamma - 2} \right) - C_2, \end{aligned}$$

where $C_2 = C \left( \frac{l t_1^{\alpha - \gamma + l - 3}}{\alpha - \gamma + l - 3} + \frac{\left( 2 + C_1 \right) t_1^{\alpha - \gamma - 1}}{\alpha - \gamma - 1} - \frac{\gamma \beta t_1^{\alpha - \gamma - 2}}{\alpha - \gamma - 2} \right)$. By Theorem 5 we have

$$\begin{aligned} E(t) \ {}&\le \ \frac{\Vert x^* \Vert ^2}{2} \frac{C \left( \frac{l t^{\alpha - \gamma + l - 3}}{\alpha - \gamma + l - 3} + \frac{\left( 2 + C_1 \right) t^{\alpha - \gamma - 1}}{\alpha - \gamma - 1} - \frac{\gamma \beta t^{\alpha - \gamma - 2}}{\alpha - \gamma - 2} \right) - C_2}{C t^{\alpha - \gamma + 1}} + \frac{C t_1^{\alpha - \gamma + 1} E(t_1)}{C t^{\alpha - \gamma + 1}} \\ {}&= \ \frac{\Vert x^* \Vert ^2}{2} \frac{\frac{l t^{\alpha - \gamma + l - 3}}{\alpha - \gamma + l - 3} + \frac{\left( 2 + C_1 \right) t^{\alpha - \gamma - 1}}{\alpha - \gamma - 1} - \frac{\gamma \beta t^{\alpha - \gamma - 2}}{\alpha - \gamma - 2}}{t^{\alpha - \gamma + 1}} + \frac{C_3}{t^{\alpha - \gamma + 1}} \\ {}&= \ \frac{\Vert x^* \Vert ^2}{2} \left( \frac{l t^{l - 4}}{\alpha - \gamma + l - 3} + \frac{\left( 2 + C_1 \right) t^{-2}}{\alpha - \gamma - 1} - \frac{\gamma \beta t^{-3}}{\alpha - \gamma - 2} \right) + \frac{C_3}{t^{\alpha - \gamma + 1}} \end{aligned}$$

where $C_3 = \frac{2C t_1^{\alpha - \gamma + 1} E(t_1) - C_2 \Vert x^* \Vert ^2}{2C}$. We know that $\frac{\alpha }{2} \le \gamma < \alpha$ and $0 \le l < 2$. Thus, in the brackets the term with $t^{-2}$ is dominating, as $t \rightarrow +\infty$. Moreover, $\alpha - \gamma + 1 > 1$. So, the behaviour of the entire expression depends on the value of $\alpha$. There exists a constant $C_4$ such that for all $t \ge t_1$

$$\begin{aligned} E(t) \ \le \ \frac{C_4}{t^2} + \frac{C_3}{t^{\alpha - \gamma + 1}}. \end{aligned}$$

That leads us to the following rates for all $t \ge t_1$

$$\begin{aligned}{} & {} \Phi _{\lambda (t)} (x(t)) - \Phi ^* \ \le \ \frac{C_4}{t^2} + \frac{C_3}{t^{\alpha - \gamma + 1}} + \frac{\Vert x^* \Vert ^2}{2t^2},\\{} & {} \Phi \left( \mathop {\textrm{prox}}\limits \nolimits _{\lambda (t) \Phi }(x(t)) \right) - \Phi ^* \ \le \ \frac{C_4}{t^2} + \frac{C_3}{t^{\alpha - \gamma + 1}} + \frac{\Vert x^* \Vert ^2}{2t^2},\\{} & {} \Vert \mathop {\textrm{prox}}\limits \nolimits _{\lambda (t) \Phi } (x(t)) - x(t) \Vert ^2 \ \le \ \frac{2C_4}{t^{2-l}} + \frac{2C_3}{t^{\alpha - \gamma - l + 1}} + \frac{\Vert x^* \Vert ^2}{t^{2-l}} \end{aligned}$$

and

$$\begin{aligned} \Vert x(t) - x_{\varepsilon (t), \lambda (t)} \Vert ^2 \ \le \ 2C_4 + \frac{2C_3}{t^{\alpha - \gamma - 1}}. \end{aligned}$$

As we can see, the strong convergence of the trajectories can no longer be shown. Nevertheless, for $C_5 = \frac{2C_4 + \Vert x^* \Vert ^2}{2}$ we deduce for all $t \ge t_1$

$$\begin{aligned}{} & {} \Phi _{\lambda (t)} (x(t)) - \Phi ^* \ \le \ \frac{C_5}{t^2} + \frac{C_3}{t^{\alpha - \gamma + 1}},\\{} & {} \Phi \left( \mathop {\textrm{prox}}\limits \nolimits _{\lambda (t) \Phi }(x(t)) \right) - \Phi ^* \ \le \ \frac{C_5}{t^2} + \frac{C_3}{t^{\alpha - \gamma + 1}} \end{aligned}$$

and

$$\begin{aligned} \Vert \mathop {\textrm{prox}}\limits \nolimits _{\lambda (t) \Phi } (x(t)) - x(t) \Vert ^2 \ \le \ \frac{2C_5}{t^{2-l}} + \frac{2C_3}{t^{\alpha - \gamma - l + 1}}. \end{aligned}$$

Since we are free to choose $\gamma$ such that $\frac{\alpha }{2} \le \gamma < \alpha$, and since we want to have as fast rates as possible, we should take $\gamma = \frac{\alpha }{2}$.

$$\begin{aligned}{} & {} \Phi _{\lambda (t)} (x(t)) - \Phi ^* \ \le \ \frac{C_5}{t^2} + \frac{C_3}{t^{\frac{\alpha }{2} + 1}},\\{} & {} \Phi \left( \mathop {\textrm{prox}}\limits \nolimits _{\lambda (t) \Phi }(x(t)) \right) - \Phi ^* \ \le \ \frac{C_5}{t^2} + \frac{C_3}{t^{\frac{\alpha }{2} + 1}} \end{aligned}$$

and

$$\begin{aligned} \Vert \mathop {\textrm{prox}}\limits \nolimits _{\lambda (t) \Phi } (x(t)) - x(t) \Vert ^2 \ \le \ \frac{2C_5}{t^{2-l}} + \frac{2C_3}{t^{\frac{\alpha }{2} - l + 1}}. \end{aligned}$$

Here we have to consider several cases.

1. If $0< \alpha < 2$, then there exists $C_6$ such that for all $t \ge t_1$
  $$\begin{aligned}{} & {} \Phi _{\lambda (t)} (x(t)) - \Phi ^* \ \le \ \frac{C_6}{t^{\frac{\alpha }{2} + 1}},\\{} & {} \Phi \left( \mathop {\textrm{prox}}\limits \nolimits _{\lambda (t) \Phi }(x(t)) \right) - \Phi ^* \ \le \ \frac{C_6}{t^{\frac{\alpha }{2} + 1}} \end{aligned}$$
  and
  $$\begin{aligned} \Vert \mathop {\textrm{prox}}\limits \nolimits _{\lambda (t) \Phi } (x(t)) - x(t) \Vert ^2 \ \le \ \frac{2C_6}{t^{\frac{\alpha }{2} - l + 1}}. \end{aligned}$$
2. If $\alpha \ge 2$, then there exists $C_6$ such that for all $t \ge t_1$
  $$\begin{aligned}{} & {} \Phi _{\lambda (t)} (x(t)) - \Phi ^* \ \le \ \frac{C_6}{t^2},\\{} & {} \Phi \left( \mathop {\textrm{prox}}\limits \nolimits _{\lambda (t) \Phi }(x(t)) \right) - \Phi ^* \ \le \ \frac{C_6}{t^2} \end{aligned}$$
  and
  $$\begin{aligned} \Vert \mathop {\textrm{prox}}\limits \nolimits _{\lambda (t) \Phi } (x(t)) - x(t) \Vert ^2 \ \le \ \frac{2C_6}{t^{2-l}}. \end{aligned}$$

$$\square $$

**Fig. 1:**
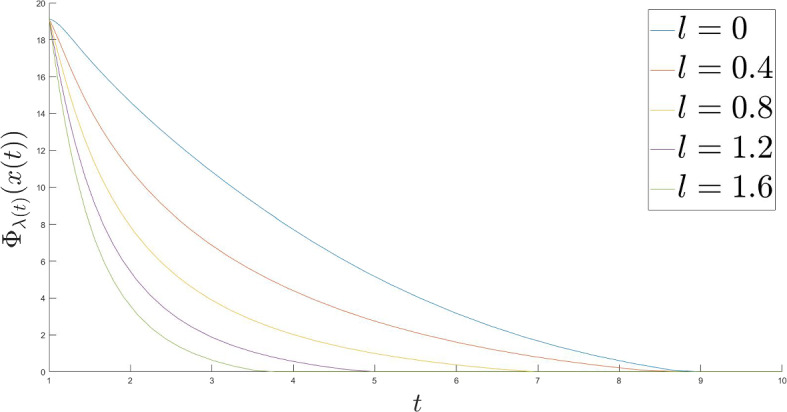
Moreau envelope values for $d = 1.9$

**Fig. 2:**
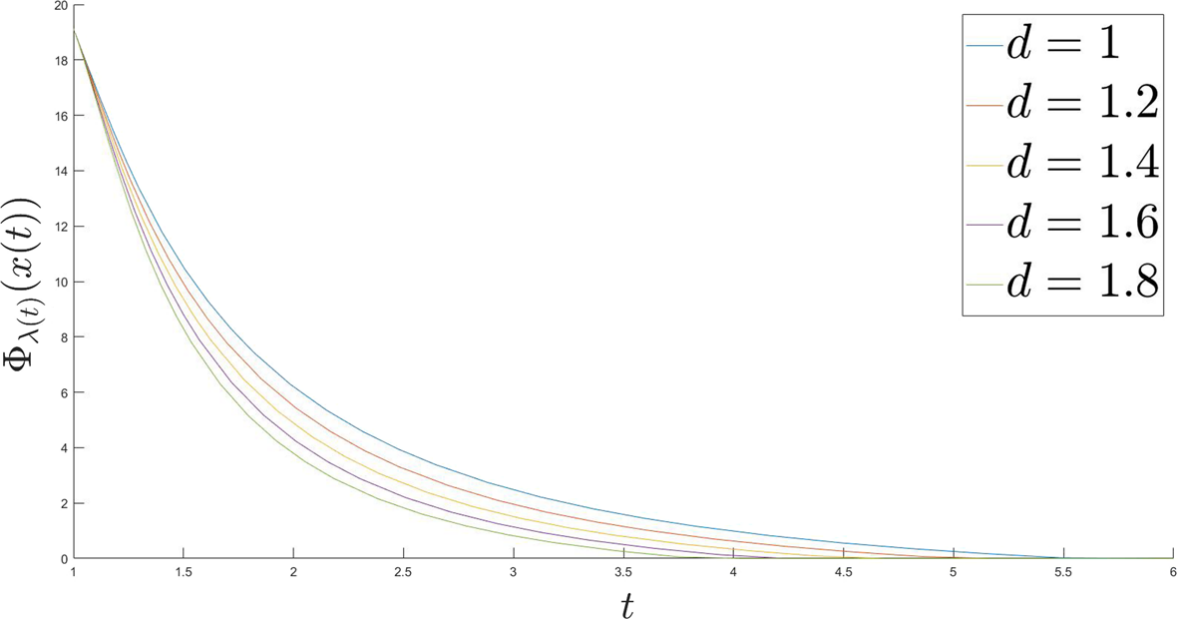
Moreau envelope values for $l = 1$

**Fig. 3:**
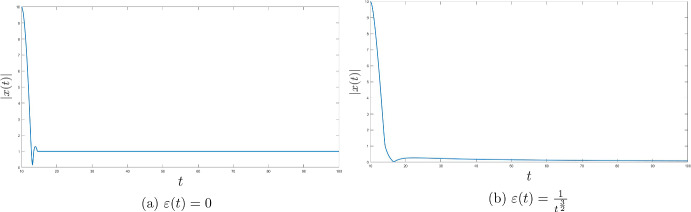
The role of Tikhonov term

**Fig. 4:**
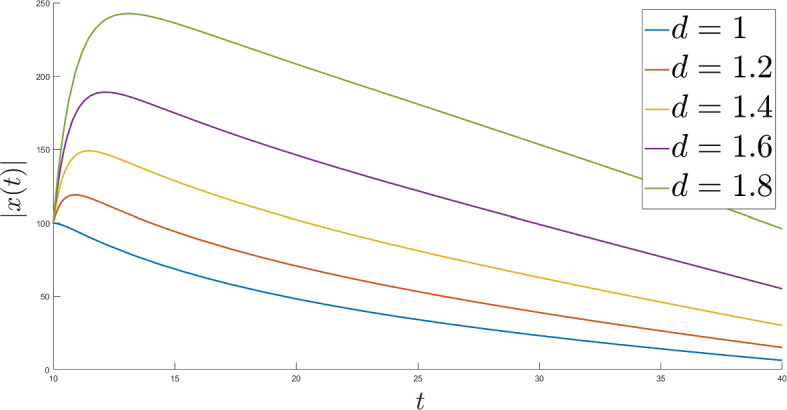
$$l = 1$$

**Fig. 5:**
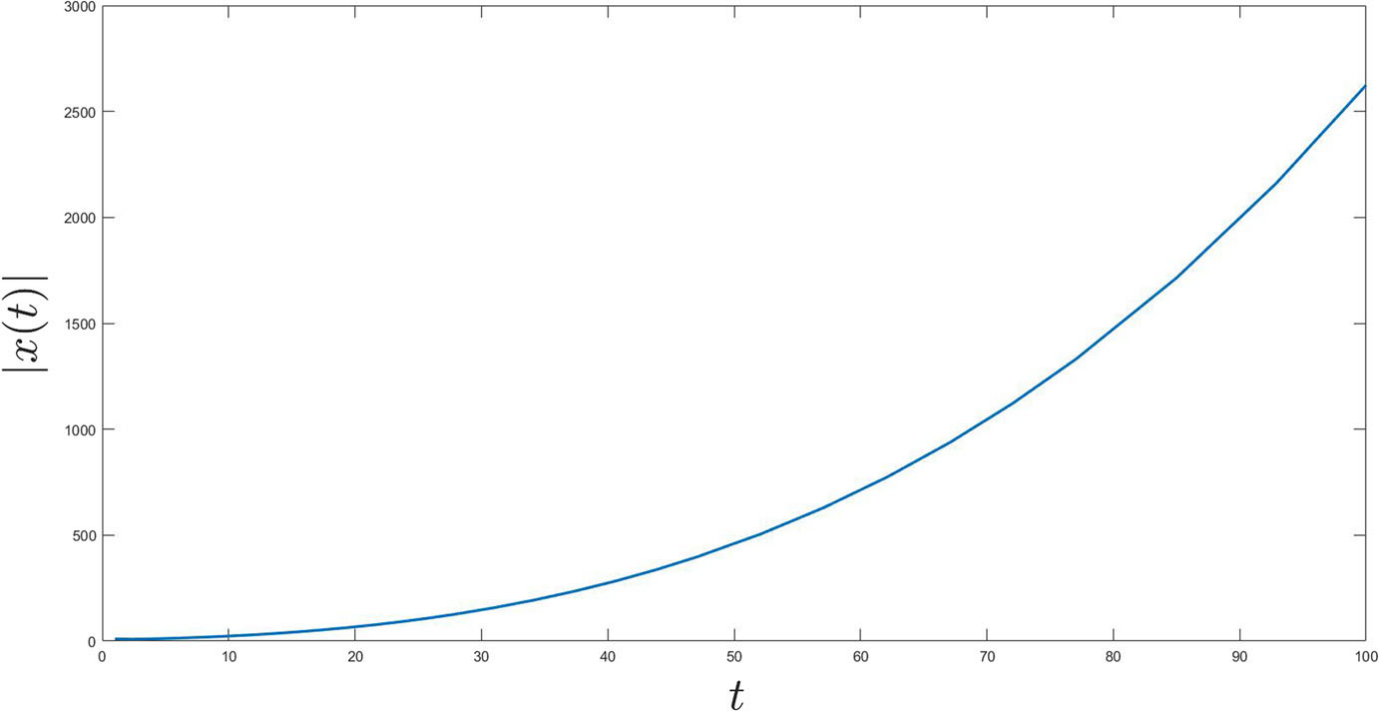
*l* and *d* do not meet the requirements

### Remark 2

Probably, it is possible to show the weak convergence of the trajectories to a minimizer of the objective function in case $d=2$.

## Numerical examples

### The rates of convergence of the Moreau envelope values

Let us consider the following objective function $\Phi : \mathbb {R} \rightarrow \mathbb {R}$, $\Phi (x) = |x| + \frac{x^2}{2}$ and plot the values of its Moreau envelope for different polynomial functions $\lambda$ and $\varepsilon$ in order to illustrate the theoretical results with some numerical examples. We set $\lambda (t) = t^l$ and $\varepsilon (t) = \frac{1}{t^d}$ with $x(t_0) = x_0 = 10$, $\dot{x}(t_0) = 0$, $\alpha = 10$, $\beta = 1$ and $t_0 = 1$.

Consider different Moreau envelope parameters $\lambda$ with $d = 1.9$ (Fig. 1):

We notice that a faster growing function $\lambda$ implies faster convergence of the Moreau envelope of the objective function $\Phi$.

Increasing the speed of decay of the Tikhonov function $\varepsilon$ for a fixed $l = 1$ accelerates the convergence of the Moreau envelope values, which was predicted by the theory (Fig. 2):

### Strong convergence of the trajectories

For the different objective function let us investigate the strong convergence of the trajectories of (1) and show some examples when the trajectories actually diverge due to one of the key assumptions of the analysis not being fulfilled. We define

$$\begin{aligned} \Phi (x) \ = \ {\left\{ \begin{array}{ll} |x - 1|, &{} \quad x > 1 \\ 0, &{} \quad x \in [-1, 1] \\ |x + 1|, &{} \quad x < -1. \end{array}\right. } \end{aligned}$$

The set $\mathop {\textrm{argmin}}\limits \Phi$ is the segment $[-1, 1]$ and clearly 0 is its element of the minimal norm. Let us investigate the influence of the Tikhonov term on the behaviour of the trajectories of the system for $\lambda (t) = t$ (Fig. 3).

As we can see in case Tikhonov function is missing the trajectories converge to a minimizer 1 of $\Phi$, however, Tikhonov term ensures the convergence to the minimal norm solution 0.

Finally, for the same choice of $\lambda$ and $\Phi$ let us take different Tikhonov functions to study their effect on the trajectories of (1). For this purpose we increase the starting point to $x(t_0) = 100$ (Fig. 4).

As we see, the faster $\varepsilon$ decays, the slower trajectories converge, which totally corresponds to the theoretical results.

To end this section let us break some of the fundamental conditions of our analysis and show that there is no convergence of the trajectories in this case (Fig. 5).

## Data Availability

In this manuscript no datasets were analysed or generated, because of the purely theoretical aspect of this research.

## Proof of the Lemma 2

### Proof

By the definition of $\varphi _{\varepsilon (t), \lambda (t)}$

$$\begin{aligned}{} & {} \varphi _{\varepsilon (t), \lambda (t)} (x_{\varepsilon (t), \lambda (t)}) = \inf _{y \in H} \left( \Phi _{\lambda (t)} (y) + \frac{\varepsilon (t)}{2} \Vert y - 0 \Vert ^2 \right) \\ {}{} & {} = \left( \Phi _{\lambda (t)} \right) _{\frac{1}{\varepsilon (t)}} (0) = \Phi _{\lambda (t) + \frac{1}{\varepsilon (t)}} (0) \end{aligned}$$

by 1 of Lemma 1. Thus,

$$\begin{aligned}{} & {} \frac{d}{dt} \left( \varphi _{\varepsilon (t), \lambda (t)} (x_{\varepsilon (t), \lambda (t)}) \right) \ = \ \frac{d}{dt} \left( \Phi _{\lambda (t) + \frac{1}{\varepsilon (t)}} (0) \right) \\ {}{} & {} = \frac{1}{2} \left( \frac{{\dot{\varepsilon }}(t)}{\varepsilon ^2(t)} - {\dot{\lambda }}(t) \right) \Vert \nabla \Phi _{\lambda (t) + \frac{1}{\varepsilon (t)}} (0) \Vert ^2, \end{aligned}$$

by (3). From (6) we obtain

$$\begin{aligned} x_{\varepsilon (t), \lambda (t)} = \mathop {\textrm{prox}}\limits \nolimits _{\frac{1}{\varepsilon (t)}\Phi _{\lambda (t)}} (0) = \frac{\mathop {\textrm{prox}}\limits \nolimits _{\left( \lambda (t) + \frac{1}{\varepsilon (t)} \right) \Phi } (0)}{\lambda (t) \varepsilon (t) + 1}, \end{aligned}$$

where the second equality comes from 2 of Lemma 1. Combining the last two equalities with

$$\begin{aligned} \Vert \mathop {\textrm{prox}}\limits \nolimits _{\lambda \Phi }(x) - \mathop {\textrm{prox}}\limits \nolimits _{\mu \Phi }(x) \Vert \le |\lambda - \mu | \Vert \nabla \Phi _{\lambda } (x)\Vert \ \forall \lambda , \ \mu > 0. \end{aligned}$$

we obtain

$$\begin{aligned} \frac{d}{dt} \left( \varphi _{\varepsilon (t), \lambda (t)} (x_{\varepsilon (t), \lambda (t)}) \right) \ {}&= \ \frac{1}{2} \left( \frac{{\dot{\varepsilon }}(t)}{\varepsilon ^2(t)} - {\dot{\lambda }}(t) \right) \left\| \frac{ 0 - \mathop {\textrm{prox}}\limits \nolimits _{\left( \lambda (t) + \frac{1}{\varepsilon (t)} \right) \Phi } (0)}{\lambda (t) + \frac{1}{\varepsilon (t)}} \right\| ^2 \\ {}&= \ \frac{1}{2} \left( \frac{{\dot{\varepsilon }}(t)}{\varepsilon ^2(t)} - {\dot{\lambda }}(t) \right) \left\| \frac{ - \left( \lambda (t) \varepsilon (t) + 1 \right) x_{\varepsilon (t), \lambda (t)}}{\lambda (t) + \frac{1}{\varepsilon (t)}} \right\| ^2 \\ {}&= \ \frac{1}{2} \left( {\dot{\varepsilon }}(t) - {\dot{\lambda }}(t) \varepsilon ^2(t) \right) \Vert x_{\varepsilon (t), \lambda (t)} \Vert ^2, \end{aligned}$$

which is the first claim.

To obtain the second claim we start with (6) noticing that for $h > 0$

$$\begin{aligned}{} & {} \nabla \Phi _{\lambda (t)} \left( x_{\varepsilon (t), \lambda (t)} \right) = -\varepsilon (t) x_{\varepsilon (t), \lambda (t)} \text { and } \nabla \Phi _{\lambda (t+h)} (x_{\varepsilon (t+h), \lambda (t+h)})\\ {}{} & {} = -\varepsilon (t+h) x_{\varepsilon (t+h), \lambda (t+h)}. \end{aligned}$$

Consider

$$\begin{aligned}&\nabla \Phi _{\lambda (t+h)} (x_{\varepsilon (t+h), \lambda (t+h)}) - \nabla \Phi _{\lambda (t)} (x_{\varepsilon (t), \lambda (t)}) \\ {}&\quad =\nabla \Phi _{\lambda (t+h)} (x_{\varepsilon (t+h), \lambda (t+h)}) - \nabla \Phi _{\lambda (t+h)} (x_{\varepsilon (t), \lambda (t)})\\ {}&\quad + \nabla \Phi _{\lambda (t+h)} (x_{\varepsilon (t), \lambda (t)}) - \nabla \Phi _{\lambda (t)} (x_{\varepsilon (t), \lambda (t)}). \end{aligned}$$

Taking the inner product of each part of this equality with $x_{\varepsilon (t+h), \lambda (t+h)} - x_{\varepsilon (t), \lambda (t)}$, we notice that

$$\begin{aligned} \langle \nabla \Phi _{\lambda (t+h)} (x_{\varepsilon (t+h), \lambda (t+h)}) - \nabla \Phi _{\lambda (t+h)} (x_{\varepsilon (t), \lambda (t)}), x_{\varepsilon (t+h), \lambda (t+h)} - x_{\varepsilon (t), \lambda (t)} \rangle \ge 0 \end{aligned}$$

by the monotonicity of $\nabla \Phi _{\lambda (t+h)}$. So,

$$\begin{aligned}&\langle \varepsilon (t) x_{\varepsilon (t), \lambda (t)} - \varepsilon (t+h) x_{\varepsilon (t+h), \lambda (t+h)}, x_{\varepsilon (t+h), \lambda (t+h)} - x_{\varepsilon (t), \lambda (t)} \rangle \ \\&\quad = \ -\varepsilon (t) \Vert x_{\varepsilon (t+h), \lambda (t+h)} - x_{\varepsilon (t), \lambda (t)} \Vert ^2 \\ {}&\qquad + \ \left( \varepsilon (t) - \varepsilon (t+h) \right) \langle x_{\varepsilon (t+h), \lambda (t+h)}, x_{\varepsilon (t+h), \lambda (t+h)} - x_{\varepsilon (t), \lambda (t)} \rangle \\ {}&\quad \ge \ \langle \nabla \Phi _{\lambda (t+h)} (x_{\varepsilon (t), \lambda (t)}) - \nabla \Phi _{\lambda (t)} (x_{\varepsilon (t), \lambda (t)}), x_{\varepsilon (t+h), \lambda (t+h)} - x_{\varepsilon (t), \lambda (t)} \rangle . \end{aligned}$$

Let us divide the last inequality by $h^2$ to obtain

$$\begin{aligned}&\frac{\varepsilon (t) - \varepsilon (t+h)}{h} \left\langle x_{\varepsilon (t+h), \lambda (t+h)}, \frac{x_{\varepsilon (t+h), \lambda (t+h)} - x_{\varepsilon (t), \lambda (t)}}{h} \right\rangle \\&\quad \ge \ \varepsilon (t) \left\| \frac{x_{\varepsilon (t+h), \lambda (t+h)} - x_{\varepsilon (t), \lambda (t)}}{h} \right\| ^2 \\ {}&\qquad + \left\langle \frac{\nabla \Phi _{\lambda (t+h)} (x_{\varepsilon (t), \lambda (t)}) - \nabla \Phi _{\lambda (t)} (x_{\varepsilon (t), \lambda (t)})}{h}, \frac{x_{\varepsilon (t+h), \lambda (t+h)} - x_{\varepsilon (t), \lambda (t)}}{h} \right\rangle . \end{aligned}$$

Now notice that, since the mapping $t \mapsto x_{\varepsilon (t), \lambda (t)}$ is Lipschitz continuous on the compact intervals of $\mathbb {R}_+ \setminus \{0\}$ (according to [1]), therefore, almost everywhere differentiable. Tending *h* to zero we deduce for almost every $t \ge t_0$

$$\begin{aligned}{} & {} -{\dot{\varepsilon }}(t) \left\langle x_{\varepsilon (t), \lambda (t)}, \frac{d}{dt} x_{\varepsilon (t), \lambda (t)} \right\rangle \ \ge \ \varepsilon (t) \left\| \frac{d}{dt} x_{\varepsilon (t), \lambda (t)} \right\| ^2 \\{} & {} \quad - \frac{2{\dot{\lambda }}(t) \Vert \nabla \Phi _{\lambda (t)} (x_{\varepsilon (t), \lambda (t)}) \Vert \left\| \frac{d}{dt} x_{\varepsilon (t), \lambda (t)} \right\| }{\lambda (t)}, \end{aligned}$$

where we used the following estimate from [12]

$$\begin{aligned}&\lim _{h \rightarrow 0} \left\langle \frac{\nabla \Phi _{\lambda (t + h)} (x_{\varepsilon (t), \lambda (t)}) - \nabla \Phi _{\lambda (t)} (x_{\varepsilon (t), \lambda (t)})}{h}, \frac{x_{\varepsilon (t+h), \lambda (t+h)} - x_{\varepsilon (t), \lambda (t)}}{h} \right\rangle \\ {}&\quad \ge - \frac{2{\dot{\lambda }}(t) \Vert \nabla \Phi _{\lambda (t)} (x_{\varepsilon (t), \lambda (t)}) \Vert \left\| \frac{d}{dt} x_{\varepsilon (t), \lambda (t)} \right\| }{\lambda (t)}. \end{aligned}$$

On the other hand, Cauchy-Schwartz inequality yields

$$\begin{aligned} -{\dot{\varepsilon }}(t) \left\langle x_{\varepsilon (t), \lambda (t)}, \frac{d}{dt} x_{\varepsilon (t), \lambda (t)} \right\rangle \le -\dot{\varepsilon }(t) \Vert x_{\varepsilon (t), \lambda (t)} \Vert \left\| \frac{d}{dt} x_{\varepsilon (t), \lambda (t)} \right\| . \end{aligned}$$

Combining the last two inequalities we arrive at

$$\begin{aligned} -{\dot{\varepsilon }}(t) \Vert x_{\varepsilon (t), \lambda (t)} \Vert + \frac{2{\dot{\lambda }}(t)}{\lambda (t)} \left\| \nabla \Phi _{\lambda (t)} (x_{\varepsilon (t), \lambda (t)}) \right\| \ \ge \ \varepsilon (t) \left\| \frac{d}{dt} x_{\varepsilon (t), \lambda (t)} \right\| . \end{aligned}$$

Replacing $\nabla \Phi _{\lambda (t)} (x_{\varepsilon (t), \lambda (t)})$ using (6) gives us the second claim. $\square$

## Proof of the Lemma 3

### Proof

Suppose that $t \ge t_0$. By the monotonicity of $\nabla \Phi _{\lambda (t)}$ we deduce

$$\begin{aligned} \left\langle \nabla \Phi _{\lambda (t)}(x_{\varepsilon (t), \lambda (t)}) - \nabla \Phi _{\lambda (t)}(x^*), x_{\varepsilon (t), \lambda (t)} - x^* \right\rangle \ \ge \ 0 \text { for all } t \ge t_0. \end{aligned}$$

By (6) we obtain

$$\begin{aligned} \left\langle - \varepsilon (t) x_{\varepsilon (t), \lambda (t)}, x_{\varepsilon (t), \lambda (t)} - x^* \right\rangle \ = \ \varepsilon (t) \left( - \Vert x_{\varepsilon (t), \lambda (t)} \Vert ^2 + \left\langle x_{\varepsilon (t), \lambda (t)}, x^* \right\rangle \right) \ \ge \ 0. \end{aligned}$$

Using Cauchy-Schwarz inequality we derive

$$\begin{aligned} \Vert x_{\varepsilon (t), \lambda (t)} \Vert \ \le \ \Vert x^* \Vert . \end{aligned}$$

This proves the first claim. For the second one consider (6) again and note that it is equivalent to

19 $$\begin{aligned} x_{\varepsilon (t), \lambda (t)} = \mathop {\textrm{prox}}\limits \nolimits _{\frac{1}{\varepsilon (t)}\Phi _{\lambda (t)}} (0) = \frac{\mathop {\textrm{prox}}\limits \nolimits _{\left( \lambda (t) + \frac{1}{\varepsilon (t)} \right) \Phi } (0)}{\lambda (t) \varepsilon (t) + 1} \end{aligned}$$

by the item 2 of Lemma 1. Note that by (7) we have $\lambda (t) + \frac{1}{\varepsilon (t)} \rightarrow +\infty$ and $\lambda (t) \varepsilon (t) + 1 \rightarrow 1$, as $t \rightarrow +\infty$. From now on the proof is inspired by Theorem 23.44 of [9]. Take $z \in \mathop {\textrm{argmin}}\limits \Phi = \mathop {\textrm{argmin}}\limits \Phi _\lambda$ for each $\lambda > 0$. From (19) and from the fact that the resolvent of maximally monotone operator is maximally monotone and firmly nonexpansive (see, for instance, Corollary 23.11(i) of [9]) and Cauchy-Schwarz inequality it follows that for all $t \ge t_0$ (note that *z* could be represented as $z = \mathop {\textrm{prox}}\limits \nolimits _{\frac{1}{\varepsilon (t)}\Phi _{\lambda (t)}} (z)$)

20 $$\begin{aligned} \left\| z - x_{\varepsilon (t), \lambda (t)} \right\| \Vert z - 0 \Vert \ \ge \ \left\langle z - x_{\varepsilon (t), \lambda (t)}, z - 0 \right\rangle \ \ge \ \Vert z - x_{\varepsilon (t), \lambda (t)} \Vert ^2, \end{aligned}$$

which gives the boundedness of $x_{\varepsilon (t), \lambda (t)}$ for all $t \ge t_0$. Now, let *y* be a weak sequential cluster point of $\{ x_{\varepsilon (t_n), \lambda (t_n)} \}_{n \in \mathbb {N}}$, namely, $x_{\varepsilon (t_{k_n}), \lambda (t_{k_n})} \rightharpoonup y$, as $n \rightarrow +\infty$. From (6) we deduce

$$\begin{aligned} \nabla \Phi _{\lambda (t_{k_n})} (x_{\varepsilon (t_{k_n}), \lambda (t_{k_n})}) + \varepsilon (t_{k_n}) x_{\varepsilon (t_{k_n}), \lambda (t_{k_n})} = 0. \end{aligned}$$

Using

$$\begin{aligned} \nabla \Phi _\lambda \ = \ \left( \partial \Phi \right) _\lambda \ = \ \frac{1}{\lambda } \left( \mathop {\textrm{Id}}\limits - \left( \mathop {\textrm{Id}}\limits + \lambda \partial \Phi \right) ^{-1} \right) \quad \forall \lambda > 0 \end{aligned}$$

we further obtain

$$\begin{aligned} \left( \mathop {\textrm{Id}}\limits + \lambda (t_{k_n}) \partial \Phi \right) ^{-1} \big ( x_{\varepsilon (t_{k_n}), \lambda (t_{k_n})} \big ) \ = \ \Big ( \lambda (t_{k_n}) \varepsilon (t_{k_n}) + 1 \Big ) x_{\varepsilon (t_{k_n}), \lambda (t_{k_n})}, \end{aligned}$$

which is equivalent to

$$\begin{aligned}{} & {} x_{\varepsilon (t_{k_n}), \lambda (t_{k_n})} \ \in \ \Big ( \lambda (t_{k_n}) \varepsilon (t_{k_n}) + 1 \Big ) x_{\varepsilon (t_{k_n}), \lambda (t_{k_n})}\\ {}{} & {} + \lambda (t_{k_n}) \partial \Phi \left( \left( \lambda (t_{k_n}) \varepsilon (t_{k_n}) + 1 \right) x_{\varepsilon (t_{k_n}), \lambda (t_{k_n})} \right) \end{aligned}$$

or

21 $$\begin{aligned} \frac{ -\varepsilon (t_{k_n}) }{\lambda (t_{k_n}) \varepsilon (t_{k_n}) + 1} x_{\varepsilon (t_{k_n}), \lambda (t_{k_n})} \ \in \ \partial \Phi \left( x_{\varepsilon (t_{k_n}), \lambda (t_{k_n})} \right) . \end{aligned}$$

The sequence

$$\begin{aligned} \left\{ x_{\varepsilon (t_{k_n}), \lambda (t_{k_n})}, \frac{ -\varepsilon (t_{k_n}) }{\lambda (t_{k_n}) \varepsilon (t_{k_n}) + 1} x_{\varepsilon (t_{k_n}), \lambda (t_{k_n})} \right\} \end{aligned}$$

lies in $\mathop {\textrm{gra}}\limits \partial \Phi$ by (21) and converges to (*y*, 0) in $H^{weak} \times H^{strong}$ due to the sequence $\{ x_{\varepsilon (t_{k_n}), \lambda (t_{k_n})} \}_{n \in \mathbb {N}}$ being also bounded and (7). Therefore, since $\mathop {\textrm{gra}}\limits \partial \Phi$ is sequentially closed (see Proposition 20.38(ii) of [9]) it follows that $y \in \mathop {\textrm{argmin}}\limits \Phi$. From (20) we derive

$$\begin{aligned} \Vert y - x_{\varepsilon (t_{k_n}), \lambda (t_{k_n})} \Vert ^2 \ \le \ \left\langle y - 0, y - x_{\varepsilon (t_{k_n}), \lambda (t_{k_n})} \right\rangle \rightarrow 0, \text { as } n \rightarrow +\infty , \end{aligned}$$

by the definition of weak convergence, thus, $x_{\varepsilon (t_{k_n}), \lambda (t_{k_n})} \rightarrow y$, as $n \rightarrow +\infty$. On the other hand, (20) leads to

$$\begin{aligned} 0 \ {}&\ge \ \Vert z - x_{\varepsilon (t_{k_n}), \lambda (t_{k_n})} \Vert ^2 - \left\langle z - x_{\varepsilon (t_{k_n}), \lambda (t_{k_n})}, z - 0 \right\rangle \\ {}&= \ \left\langle z - x_{\varepsilon (t_{k_n}), \lambda (t_{k_n})}, 0 - x_{\varepsilon (t_{k_n}), \lambda (t_{k_n})} \right\rangle \rightarrow \left\langle z - y, 0 - y \right\rangle \end{aligned}$$

and thus $y = x^*$ by the characterization of $x^*$, namely,

$$\begin{aligned} \text {for } x^* \in \mathop {\textrm{argmin}}\limits \Phi \text { and } \forall z \in \mathop {\textrm{argmin}}\limits \Phi \text { it holds that } \langle z - x^*, 0 - x^* \rangle \le 0. \end{aligned}$$

So, $x^*$ being the only weak sequential cluster point of the bounded sequence $\left\{ x_{\varepsilon (t_n), \lambda (t_n)} \right\} _{n \in \mathbb {N}}$ means that $x_{\varepsilon (t_n), \lambda (t_n)} \rightharpoonup x^*$, as $n \rightarrow +\infty$, by Lemma 2.46 of [9]. By (20) again we deduce

$$\begin{aligned} \Vert x_{\varepsilon (t_n), \lambda (t_n)} - x^* \Vert ^2 \ \le \ \left\langle 0 - x^*, x_{\varepsilon (t_n), \lambda (t_n)} - x^* \right\rangle \rightarrow 0, \text { as } n \rightarrow +\infty \end{aligned}$$

and so the second claim follows. $\square$

## Proof of the Theorem 7

### Proof

Let us notice that we can simplify a bit the proof due to

$$\begin{aligned}&\int _{t_1}^t \left( {\dot{\lambda }}(s) \varepsilon ^2(s) - \dot{\varepsilon }(s) + \frac{\gamma \beta {\dot{\varepsilon }}(s) \sqrt{\varepsilon (s)}}{2} + \gamma (2a + c \gamma ) \sqrt{\varepsilon (s)} \left( \frac{2 {\dot{\lambda }}(s)}{\lambda (s)} - \frac{{\dot{\varepsilon }}(s)}{\varepsilon (s)} \right) ^2 \right) \Gamma (s) ds \\ {}&\quad \le \int _{t_1}^t \left( {\dot{\lambda }}(s) \varepsilon ^2(s) - {\dot{\varepsilon }}(s) + \gamma (2a + c \gamma ) \sqrt{\varepsilon (s)} \left( \frac{2 {\dot{\lambda }}(s)}{\lambda (s)} - \frac{{\dot{\varepsilon }}(s)}{\varepsilon (s)} \right) ^2 \right) \Gamma (s) ds. \end{aligned}$$

The proof of the theorem will be divided into several steps.

## The asymptotic behaviour of the function $\Gamma$

Let us start with the function $\Gamma (t) \ = \ \exp \left( \int _{t_1}^t \mu (s) ds \right)$ for $\mu (t) = -\frac{\dot{\varepsilon }(t)}{2 \varepsilon (t)} + \left( \alpha - \gamma \right) \sqrt{\varepsilon (t)}$.

$$\begin{aligned} \Gamma (t) \ {}&= \ \exp \left( -\frac{1}{2} \int _{t_1}^t \frac{\dot{\varepsilon }(s)}{\varepsilon (s)} ds + (\alpha - \gamma ) \int _{t_1}^t \sqrt{\varepsilon (s)} ds \right) \ \\&= \ \exp \left( \frac{1}{2} \ln \frac{\varepsilon (t_1)}{\varepsilon (t)} + (\alpha - \gamma ) \int _{t_1}^t \sqrt{\varepsilon (s)} ds \right) \\ {}&= \ \sqrt{\frac{\varepsilon (t_1)}{\varepsilon (t)}} \exp \left( (\alpha - \gamma ) \int _{t_1}^t \sqrt{\varepsilon (s)} ds \right) . \end{aligned}$$

Since $\varepsilon (t)$ is positive for all $t \ge t_1 \ge t_0$, the integral is nonnegative and the whole exponent is lower bounded by 1. Using the property of Tikhonov function, namely, $\lim _{t \rightarrow +\infty } \varepsilon (t) = 0$, we deduce that

$$\begin{aligned} \Gamma (t) \ \ge \ \sqrt{\frac{\varepsilon (t_1)}{\varepsilon (t)}} \rightarrow +\infty \text { as } t \rightarrow +\infty . \end{aligned}$$

## The asymptotic behaviour of the function *E*

For now it is enough to assume the following, but later we will have to strengthen these assumptions:

22 $$\begin{aligned} \begin{aligned}&\lim _{t \rightarrow +\infty } {\dot{\lambda }}(t) \varepsilon ^\frac{3}{2}(t) \ = \ 0 \text { and } \\ {}&\lim _{t \rightarrow +\infty } \frac{\dot{\lambda }(t)}{\lambda (t)} \ = \ 0. \end{aligned} \end{aligned}$$

Let us recall the form of the energy function

$$\begin{aligned} E(t)&= \varphi _{\varepsilon (t), \lambda (t)} (x(t)) - \varphi _{\varepsilon (t), \lambda (t)} (x_{\varepsilon (t), \lambda (t)}) + \frac{1}{2} \left\| \gamma \sqrt{\varepsilon (t)} \left( x(t) - x_{\varepsilon (t), \lambda (t)} \right) \right. \\ {}&\quad \left. + \dot{x}(t) + \beta \nabla \Phi _{\lambda (t)} (x(t)) \right\| ^2, \end{aligned}$$

where $\frac{\alpha }{2} \le \gamma < \alpha$. Let us study the behaviour of the function

$$\begin{aligned}{} & {} \frac{\int _{t_1}^t \left[ \left( {\dot{\lambda }}(s) \varepsilon ^2(s) - {\dot{\varepsilon }}(s) + \frac{\big ( 2 {\dot{\lambda }}(s) \varepsilon (s) - \lambda (s) {\dot{\varepsilon }}(s) \big )^2 (2a + c \gamma ) \gamma }{\lambda ^2(s) \varepsilon ^{\frac{3}{2}}(s)} \right) \exp \left( \int _{t_1}^s \mu (u) du \right) \right] ds}{\exp \left( \int _{t_1}^t \mu (u) du \right) } \\{} & {} \quad = \ \frac{\int _{t_1}^t \tilde{g}(s) \Gamma (s) ds}{\Gamma (t)}, \end{aligned}$$

as $t \rightarrow +\infty$. Since $\tilde{g}(t) \Gamma (t) \ \ge \ 0$ for all $t \ge t_1$ so is the integral $\int _{t_1}^t \tilde{g}(s) \Gamma (s) ds$. If $0 \ \le \ \int _{t_1}^t \tilde{g}(s) \Gamma (s) ds \ \le \ +\infty$, then *E*(*t*) goes to zero as $t \rightarrow +\infty$ due to the properties of *h* and Theorem 6. Otherwise, we may apply L’Hospital’s rule to obtain

$$\begin{aligned}&\lim _{t \rightarrow +\infty } \frac{\int _{t_1}^t \tilde{g}(s) \Gamma (s) ds}{\Gamma (t)} \ = \ \lim _{t \rightarrow +\infty } \frac{\tilde{g}(t)}{\mu (t)} \\&\quad = \lim _{t \rightarrow +\infty } \left( \frac{2 {\dot{\lambda }}(t) \varepsilon ^3(t) - 2 \varepsilon (t) {\dot{\varepsilon }}(t)}{2(\alpha - \gamma ) \varepsilon ^\frac{3}{2}(t) - {\dot{\varepsilon }}(t)} + \frac{2 \varepsilon (t) \big ( 2 {\dot{\lambda }}(t) \varepsilon (t) - \lambda (t) {\dot{\varepsilon }}(t) \big )^2 (a + c \gamma ) \gamma }{\lambda ^2(t) \varepsilon ^{\frac{3}{2}}(t) \left( 2(\alpha - \gamma ) \varepsilon ^\frac{3}{2}(t) - {\dot{\varepsilon }}(t) \right) } \right) , \end{aligned}$$

if the latest exists, which we are going to show now. Consider

$$\begin{aligned} \frac{2 {\dot{\lambda }}(t) \varepsilon ^3(t) - 2 \varepsilon (t) \dot{\varepsilon }(t)}{2(\alpha - \gamma ) \varepsilon ^\frac{3}{2}(t) - \dot{\varepsilon }(t)} \ = \ 2 \frac{{\dot{\lambda }}(t) \varepsilon ^\frac{3}{2}(t) - \frac{\dot{\varepsilon }(t)}{\sqrt{\varepsilon (t)}}}{2(\alpha - \gamma ) - \frac{{\dot{\varepsilon }}(t)}{\varepsilon ^\frac{3}{2}(t)}} \ \le \ \frac{{\dot{\lambda }}(t) \varepsilon ^\frac{3}{2}(t) - \frac{\dot{\varepsilon }(t)}{\sqrt{\varepsilon (t)}}}{\alpha - \gamma }, \end{aligned}$$

since $- \frac{{\dot{\varepsilon }}(t)}{\varepsilon ^\frac{3}{2}(t)} \ge 0$. Notice that

$$\begin{aligned}{} & {} \lim _{t \rightarrow +\infty } \frac{-\dot{\varepsilon }(t)}{\sqrt{\varepsilon (t)}} \ = \ 0 \text { by } (11), \text { since } 0 \\ {}{} & {} \quad \le \ -\frac{\dot{\varepsilon }(t)}{\sqrt{\varepsilon (t)}} \ \le \ 2 \left( \alpha - \gamma \frac{a + 1}{a} \right) \varepsilon (t) \rightarrow 0, \text { as } t \rightarrow +\infty . \end{aligned}$$

So, by (22) we deduce

$$\begin{aligned} \lim _{t \rightarrow +\infty } \frac{2 {\dot{\lambda }}(t) \varepsilon ^3(t) - 2 \varepsilon (t) {\dot{\varepsilon }}(t)}{2(\alpha - \gamma ) \varepsilon ^\frac{3}{2}(t) - {\dot{\varepsilon }}(t)} \ = \ 0. \end{aligned}$$

Consider now

$$\begin{aligned} \frac{2 \big ( 2 {\dot{\lambda }}(t) \varepsilon (t) - \lambda (t) \dot{\varepsilon }(t) \big )^2 (2a + c \gamma ) \gamma }{\lambda ^2(t) \sqrt{\varepsilon (t)} \left( 2(\alpha - \gamma ) \varepsilon ^\frac{3}{2}(t) - {\dot{\varepsilon }}(t) \right) } \ {}&\le \ \frac{2 \big ( 2 {\dot{\lambda }}(t) \varepsilon (t) - \lambda (t) \dot{\varepsilon }(t) \big )^2 (2a + c \gamma ) \gamma }{2(\alpha - \gamma ) \lambda ^2(t) \varepsilon ^2(t)} \\ {}&= \ \frac{(2a + c \gamma ) \gamma }{\alpha - \gamma } \left( \frac{2 {\dot{\lambda }}(t)}{\lambda (t)} - \frac{{\dot{\varepsilon }}(t)}{\varepsilon (t)} \right) ^2. \end{aligned}$$

Again, by (11) we know that

$$\begin{aligned} 0 \ \le \ -\frac{{\dot{\varepsilon }}(t)}{\varepsilon (t)} \ \le \ 2 \left( \alpha - \gamma \frac{a + 1}{a} \right) \sqrt{\varepsilon (t)} \rightarrow 0, \text { as } t \rightarrow +\infty . \end{aligned}$$

So, again using (22) we deduce that $\lim _{t \rightarrow +\infty } E(t) \ = \ 0$.

## The asymptotic behaviour of the function $\frac{E}{\varepsilon }$

For this part we assume the full set of conditions (17). In the same spirit let us analyse the asymptotic behaviour of $\frac{E(t)}{\varepsilon (t)}$ as $t \rightarrow +\infty$. From Theorem 6 we know that

$$\begin{aligned} \frac{E(t)}{\varepsilon (t)} \le \frac{\int _{t_1}^t \tilde{g}(s) \Gamma (s) ds}{\varepsilon (t) \Gamma (t)} \Vert x^* \Vert + \frac{E(t_1)}{\varepsilon (t) \Gamma (t)}. \end{aligned}$$

By (17) we immediately deduce that $\lim _{t \rightarrow +\infty } \frac{E(t_1)}{\varepsilon (t) \Gamma (t)} = 0$. For the first term let us use the same technique as in the previous chapter and apply L’Hospital’s rule to obtain

$$\begin{aligned}&\lim _{t \rightarrow +\infty } \frac{\int _{t_1}^t \tilde{g}(s) \Gamma (s) ds}{\varepsilon (t) \Gamma (t)} \ = \ \lim _{t \rightarrow +\infty } \frac{\tilde{g}(t) \Gamma (t)}{{\dot{\varepsilon }}(t) \Gamma (t) + \mu (t) \varepsilon (t) \Gamma (t)} \ = \ \lim _{t \rightarrow +\infty } \frac{\tilde{g}(t)}{{\dot{\varepsilon }}(t) + \mu (t) \varepsilon (t)} \\&\quad = \lim _{t \rightarrow +\infty } \left( {\dot{\lambda }}(t) \varepsilon ^2(t) - {\dot{\varepsilon }}(t) + \frac{\big ( 2 {\dot{\lambda }}(t) \varepsilon (t) - \lambda (t) {\dot{\varepsilon }}(t) \big )^2 (2a + c \gamma ) \gamma }{\lambda ^2(t) \varepsilon ^{\frac{3}{2}}(t)} \right) \frac{1}{{\dot{\varepsilon }}(t) + \mu (t) \varepsilon (t)} \\ {}&\quad = \lim _{t \rightarrow +\infty } \left( {\dot{\lambda }}(t) \varepsilon ^2(t) - \dot{\varepsilon }(t) + \frac{\big ( 2 {\dot{\lambda }}(t) \varepsilon (t) - \lambda (t) {\dot{\varepsilon }}(t) \big )^2 (2a + c \gamma ) \gamma }{\lambda ^2(t) \varepsilon ^{\frac{3}{2}}(t)} \right) \frac{1}{\frac{{\dot{\varepsilon }}(t)}{2} + (\alpha - \gamma ) \varepsilon ^\frac{3}{2}(t)} \\ {}&\quad = \lim _{t \rightarrow +\infty } \left( \frac{{\dot{\lambda }}(t) \varepsilon ^2(t) - \dot{\varepsilon }(t)}{\frac{{\dot{\varepsilon }}(t)}{2} + (\alpha - \gamma ) \varepsilon ^\frac{3}{2}(t)} + \frac{\big ( 2 {\dot{\lambda }}(t) \varepsilon (t) - \lambda (t) {\dot{\varepsilon }}(t) \big )^2 (2a + c \gamma ) \gamma }{\lambda ^2(t) \varepsilon ^{\frac{3}{2}}(t) \left( \frac{{\dot{\varepsilon }}(t)}{2} + (\alpha - \gamma ) \varepsilon ^\frac{3}{2}(t) \right) } \right) \\ {}&\quad = \lim _{t \rightarrow +\infty } \frac{{\dot{\lambda }}(t) \lambda ^2(t) \varepsilon ^{\frac{7}{2}}(t) - {\dot{\varepsilon }}(t) \lambda ^2(t) \varepsilon ^{\frac{3}{2}}(t) + \big ( 2 {\dot{\lambda }}(t) \varepsilon (t) - \lambda (t) {\dot{\varepsilon }}(t) \big )^2 (2a + c \gamma ) \gamma }{\frac{\lambda ^2(t) \varepsilon ^{\frac{3}{2}}(t) \dot{\varepsilon }(t)}{2} + (\alpha - \gamma ) \lambda ^2(t) \varepsilon ^3(t)} \\ {}&\quad = \lim _{t \rightarrow +\infty } \frac{{\dot{\lambda }}(t) \sqrt{\varepsilon (t)} - \frac{\dot{\varepsilon }(t)}{\varepsilon ^{\frac{3}{2}}(t)} + \left( \frac{2 \dot{\lambda }(t) \varepsilon (t) - \lambda (t) \dot{\varepsilon }(t)}{\lambda (t) \varepsilon ^\frac{3}{2}(t)} \right) ^2 (2a + c \gamma ) \gamma }{\frac{{\dot{\varepsilon }}(t)}{2 \varepsilon ^{\frac{3}{2}}(t) } + \alpha - \gamma } \ = \ 0 \text { by } (17). \end{aligned}$$

Thus, we have established that $\lim _{t \rightarrow +\infty } \frac{E(t)}{\varepsilon (t)} \ = \ 0$.

## The asymptotic behaviour of the function $\lambda E$

In this section we need to assume the full set of conditions (17) again. We will study the behaviour of $\lambda (t) E(t)$, as $t \rightarrow +\infty$. Again, from Theorem 6 we know that

$$\begin{aligned} \lambda (t) E(t) \le \frac{\lambda (t) \int _{t_1}^t \tilde{g}(s) \Gamma (s) ds}{\Gamma (t)} \Vert x^* \Vert + \frac{E(t_1) \lambda (t)}{\Gamma (t)}. \end{aligned}$$

We immediately obtain that $\frac{E(t_1) \lambda (t)}{\Gamma (t)} \rightarrow 0$ as $t \rightarrow +\infty$, since

$$\begin{aligned}{} & {} \lim _{t \rightarrow +\infty } \frac{\exp \left( (\alpha - \gamma ) \int _{t_1}^t \sqrt{\varepsilon (s)} ds \right) }{\lambda (t) \sqrt{\varepsilon (t)}} \ = \ \lim _{t \rightarrow +\infty } \frac{\sqrt{\varepsilon (t)} \exp \left( (\alpha - \gamma ) \int _{t_1}^t \sqrt{\varepsilon (s)} ds \right) }{\lambda (t) \varepsilon (t)} \\ {}{} & {} \quad = \ +\infty \text { by } (7) \text { and } (17). \end{aligned}$$

Arguing in the same way we deduce for the first term

$$\begin{aligned} \lim _{t \rightarrow +\infty } \frac{\int _{t_1}^t \tilde{g}(s) \Gamma (s) ds}{\frac{\Gamma (t)}{\lambda (t)}} \ = \ \lim _{t \rightarrow +\infty } \frac{\tilde{g}(t) \Gamma (t)}{\frac{\mu (t) \Gamma (t) \lambda (t) - \Gamma (t) {\dot{\lambda }}(t)}{\lambda ^2(t)}} \ = \ \lim _{t \rightarrow +\infty } \frac{\tilde{g}(t) \lambda ^2(t)}{\mu (t) \lambda (t) - \dot{\lambda }(t)}. \end{aligned}$$

Consider

$$\begin{aligned}&\frac{\tilde{g}(t) \lambda ^2(t)}{\mu (t) \lambda (t) - \dot{\lambda }(t)} = \frac{\left( {\dot{\lambda }}(t) \varepsilon ^2(t) - {\dot{\varepsilon }}(t) + \frac{\big ( 2 {\dot{\lambda }}(t) \varepsilon (t) - \lambda (t) {\dot{\varepsilon }}(t) \big )^2 (2a + c \gamma ) \gamma }{\lambda ^2(t) \varepsilon ^{\frac{3}{2}}(t)} \right) \lambda ^2(t)}{-\frac{{\dot{\varepsilon }}(t) \lambda (t)}{2 \varepsilon (t)} + \left( \alpha - \gamma \right) \sqrt{\varepsilon (t)} \lambda (t) - {\dot{\lambda }}(t)} \\ {}&\quad = 2\frac{{\dot{\lambda }}(t) \varepsilon ^3(t) \sqrt{\varepsilon (t)} \lambda ^2(t) - {\dot{\varepsilon }}(t) \varepsilon (t) \sqrt{\varepsilon (t)} \lambda ^2(t) + \big ( 2 {\dot{\lambda }}(t) \varepsilon (t) - \lambda (t) {\dot{\varepsilon }}(t) \big )^2 (2a + c \gamma ) \gamma }{-{\dot{\varepsilon }}(t) \sqrt{\varepsilon (t)} \lambda (t) + 2 \left( \alpha - \gamma \right) \varepsilon ^2(t) \lambda (t) - 2 {\dot{\lambda }}(t) \varepsilon (t) \sqrt{\varepsilon (t)}} \\ {}&\quad = 2\frac{{\dot{\lambda }}(t) \varepsilon ^\frac{3}{2}(t) \lambda (t) - \frac{{\dot{\varepsilon }}(t) \lambda (t)}{\sqrt{\varepsilon (t)}} + \left( \frac{2 {\dot{\lambda }}(t) \varepsilon (t) - \lambda (t) \dot{\varepsilon }(t)}{\varepsilon (t) \sqrt{\lambda (t)}} \right) ^2 (2a + c \gamma ) \gamma }{\frac{-\dot{\varepsilon }(t)}{\varepsilon ^\frac{3}{2}(t)} + 2 \left( \alpha - \gamma \right) - \frac{2 {\dot{\lambda }}(t)}{\lambda (t) \sqrt{\varepsilon (t)}}} \rightarrow 0 \text { as } t \rightarrow \\&\qquad +\infty \text { by } (7), (11) \text { and } (17), \end{aligned}$$

since

$$\begin{aligned}{} & {} 0 \ \le \ {\dot{\lambda }}(t) \varepsilon ^\frac{3}{2}(t) \lambda (t) \ = \ {\dot{\lambda }}(t) \sqrt{\varepsilon (t)} \varepsilon (t) \lambda (t) \rightarrow 0, \text { as } t \rightarrow +\infty ,\\{} & {} 0 \ \le \ \frac{-{\dot{\varepsilon }}(t) \lambda (t)}{\sqrt{\varepsilon (t)}} \ \le \ 2 \left( \alpha - \gamma \frac{a + 1}{a} \right) \lambda (t) \varepsilon (t) \rightarrow 0, \text { as } t \rightarrow +\infty ,\\{} & {} 0 \ \le \ \frac{-{\dot{\varepsilon }}(t) \sqrt{\lambda (t)}}{\varepsilon (t)} \ \le \ 2 \left( \alpha - \gamma \frac{a + 1}{a} \right) \sqrt{\varepsilon (t) \lambda (t)} \rightarrow 0, \text { as } t \rightarrow +\infty \end{aligned}$$

and

$$\begin{aligned} \frac{{\dot{\lambda }}(t)}{\sqrt{\lambda (t)}} \rightarrow 0, \text { as } t \rightarrow +\infty . \end{aligned}$$

Thus, we have established that $\lim _{t \rightarrow +\infty } \frac{E(t)}{\lambda (t)} \ = \ 0$. $\square$

## Polynomial choice of parameters satisfies the key assumptions (7) and (11)–(14)

The set of assumptions for $\lambda (t) = t^l$ and $\varepsilon (t) = \frac{1}{t^d}$, *l*, $d > 0$ becomes

(i) $\lim _{t \rightarrow +\infty } t^{l-d} \ = \ 0$; There exist $\frac{\alpha }{2} \le \gamma < \alpha , \ a> 0 \text { and } c > 0$ such that for all *t* large enough
(ii) $$ \frac{d}{2} t^{\frac{d}{2}-1} \ \le \ \min \left\{ 2 \gamma - \alpha + \frac{\gamma \beta d}{2t}, \ \alpha - \gamma \frac{a + 1}{a} \right\} $$;
(iii) $$ \left( 2 \gamma (\alpha - \gamma ) + \frac{\gamma }{c} - 1 \right) \frac{1}{t^d} + \frac{d \beta }{t^{d+1}} \ \le \ 0 $$;
(iv) $$ \frac{2 \beta }{t^{2d}} - d \left( 2 - \frac{\gamma \beta }{t^\frac{d}{2}} \right) \frac{1}{t^{d+1}} \ \le \ 0 $$ and
(v) $$ \left( \frac{\gamma }{a} + 2 (\alpha - \gamma ) \right) \beta ^2 \frac{1}{t^\frac{d}{2}} + \frac{3d \beta ^2}{2 t} - l t^{l-1} \ \le \ \beta $$.

The conditions above are, in turn, equivalent to

(i) $$ l < d $$;
(ii) $$d \le 2$$;
(iii) $$ 2 \gamma (\alpha - \gamma ) \ < \ 1 $$;
(iv) $$d \ge 1$$ and
(v) is always satisfied starting from *t* large enough.

Finally, we deduce for *l* and *d*

$$\begin{aligned}&1 \le d \le 2, \\&0 \le l < d, \end{aligned}$$

which is our desired setting to satisfy all the conditions and to guarantee that the main results of this paper are valid.

### Remark 3

Condition (iii) does not contradict with the choice of $\gamma$, namely, $\gamma$ could be chosen to satisfy both of them at the same time:

$$\begin{aligned} 2 \gamma (\alpha - \gamma ) \,< \ 1 \text { and } \frac{\alpha }{2} \le \gamma < \alpha . \end{aligned}$$

Indeed, (iii) implies

$$\begin{aligned} \gamma ^2 - \alpha \gamma + \frac{1}{2} > 0. \end{aligned}$$

If $\alpha < \sqrt{2}$, then $\gamma ^2 - \alpha \gamma + \frac{1}{2}$ is always positive and we are free to choose $\gamma$ such that $\frac{\alpha }{2} \le \gamma < \alpha$. Otherwise, $\alpha \ge \sqrt{2}$ means that

$$\begin{aligned} \gamma \ < \ \frac{\alpha - \sqrt{\alpha ^2 - 2}}{2} \text { or } \gamma \ > \ \frac{\alpha + \sqrt{\alpha ^2 - 2}}{2} \end{aligned}$$

and thus we take

$$\begin{aligned} \frac{\alpha }{2} \ \le \ \frac{\alpha + \sqrt{\alpha ^2 - 2}}{2} \,< \ \gamma \, < \alpha . \end{aligned}$$
